# Microwave-Assisted Extraction of Bioactive Compounds from Mandarin Peel: A Comprehensive Biorefinery Strategy

**DOI:** 10.3390/antiox14060722

**Published:** 2025-06-12

**Authors:** Marina Jurić, Nikolina Golub, Emerik Galić, Kristina Radić, Luna Maslov Bandić, Dubravka Vitali Čepo

**Affiliations:** 1 Department of Nutrition and Dietetics, University of Zagreb Faculty of Pharmacy and Biochemistry, Ante Kovačića 1, 10000 Zagreb, Croatia; marina.juric@pharma.unizg.hr (M.J.); emerik.galic@pharma.unizg.hr (E.G.); kristina.radic@pharma.unizg.hr (K.R.); 2 Department of Chemistry, University of Zagreb Faculty of Agriculture, Svetošimunska 25, 10000 Zagreb, Croatia; lmaslov@agr.hr

**Keywords:** mandarin peel, response surface methodology, carotenoids, polyphenols, pectin, biorefinery approach, microwave-assisted extraction

## Abstract

Mandarin peel is a valuable but underutilized source of numerous bioactive compounds. The main focus of this work was to develop an integrated two-step process for the subsequent extraction of polyphenols and carotenoids (step 1) and pectin (step 2) from mandarin peel by microwave-assisted extraction (MAE), using only green solvents. This represents a novel, scale-up-suitable approach that might contribute to the improved revalorization of mandarin peel. Response surface methodology was used to maximize the yields of polyphenols, flavonoids, and carotenoids, as well as antioxidative activity (DPPH- and ABTS-radical scavenging capacity). The sample-to-solvent ratio and solvent type significantly influenced extractability of polyphenols and carotenoids, while extraction time and power were the key variables influencing pectin yield. Optimal extracts contained 21.76 ± 0.46 mg GAE/g of polyphenols (with 139.7 ± 2.28 mg/g and 703.62 ± 51.72 µg/g of tangeretin and nobiletin, respectively); 352.3 ± 17.4 µg/g of β-carotene and 273 ± 23 mg/g of pectin. MAE resulted in either higher yields, reduced extraction times or both, compared to conventional solvent extraction (CSE), depending on the target compound. The energy consumption of MAE was considerably lower, compared to CSE, in four out of five developed extraction procedures. Pectin obtained in the integrated two-step process had higher purity compared to pectin extracted from intact mandarin peel.

## 1. Introduction

More than 120 million tonnes of mandarins (*Citrus reticulata* Blanco) are produced worldwide [1], and during processing 50–60% of the whole fruit remains as a by-product (peels, pomace, and seeds). In Croatia, the average annual mandarin production between 2020 and 2024 was 38,981 tonnes [2]. Considering that Medved et al. obtained 72 g of mandarin peel powder from 1 kg of mandarin fruit, it can be estimated that Croatia produces around 2807 tonnes of mandarin peel annually [3].

Mandarin peel is rich in bioactive components such as polyphenols, flavonoids, carotenoids and pectin [4], with a huge market for application in the pharma and food industries. For example, in 2022, the global polyphenol market was estimated to be worth USD 1.68 billion. From 2023 to 2030, it is projected to expand at a compound annual growth rate of 7.4% [5]. Similar information has been provided by another report [6], which projected a market size of USD 2.5 billion by 2030 at a 7.5% annual growth rate. Pectin, on the other hand, is frequently employed as a gelling, stabilizing, emulsifying and thickening ingredient in food and pharmaceutical goods [7]. The pectin market was worth USD 1.31 billion in 2023 and is expected to grow at 6.9% up to 2032 [8]. The carotenoid market will be worth USD 9 billion by 2031, with an expected annual growth of 4.5% [9].

In view of attempts to enhance the sustainable recovery of valuable bioactive compounds from natural sources, the contemporary focus of scientific investigations is set on the development and application of innovative green extraction techniques. Among them, microwave-assisted extraction (MAE) offers significant advantages compared to conventional solvent extraction (CSE)—it is considered a reliable and safe method resulting in higher extraction yields, reduced solvent use and shorter time of extraction. Additionally, it leads to lower rates of oxidation and degradation of bioactive compounds of interest [10]. Importantly, MAE technology has matured to the point of industrial scale-up, with commercially available continuous-flow systems, thus supporting its economic feasibility and technological readiness for large-scale applications. This combination of sustainability, efficiency and scalability positions MAE as a promising alternative for the extraction of natural products in food, pharmaceutical and cosmetic industries.

Even though investigations focused on the valorisation of mandarin peel as the source of polyphenols are numerous, the application of MAE for this purpose has been investigated by only several researchers. Kaur et al. [11] applied methanol, ethanol, acetonitrile, ethyl acetate and hexane, obtaining the highest phenolic content and antioxidant activity with 80% ethanol. Similarly, Inoue et al. [12] and Nishad et al. [13] obtained the highest yields using 70% ethanol, while Hayat et al. [14] applied 80% methanol. It is important to emphasize that in the mentioned investigations, in-house fabricated microwave extraction systems were applied for extraction. Compared to this approach, the utilization of laboratory closed-vessel systems for MAE offers numerous advantages. It provides a wider range of microwave power and wider choice of solvents; prevents solvent evaporation and thus reduces solvent use; prevents overpressure incidents and enables uniform heating, which altogether might result in higher yields and lower environmental impact of the process [15]. It is also important to emphasize that, to our knowledge, there is no available research that specifically targeted MAE yields of the most abundant polyphenolic compounds in mandarin peel—flavonoids nobiletin and tangeretin [16]. They show protective effects against memory impairment in Alzheimer’s disease, dementia and Parkinson’s disease, so their content is particularly important in terms of obtaining functional mandarin-peel based polyphenol extracts [17,18].

The most abundant carotenoids in mandarin peel are β-cryptoxanthin, β-citraurin and violaxanthin, with β-cryptoxanthin often dominating (62.1% of total carotenoids in some varieties) [19]. Available research shows that the extraction of carotenoids from mandarin peel is usually conducted by utilizing organic solvents, such as hexane or acetone, and highlights ultrasound-assisted extraction (UAE) as more effective compared to CSE [20,21]. To our knowledge, there are no available investigations on the application of MAE for carotenoids from mandarin peel, and studies on the possibilities of green extraction solvents are limited. Anticona et al. [21] applied ethanol–water mixtures for UAE of antioxidants (including carotenoids) from mandarin peel while Maslov Bandić et al. [22] optimized solid–liquid extraction of carotenoids using limonene.

Both polyphenols and carotenoids are recognized as potential antioxidants; however, they act through distinct mechanisms. Polyphenols primarily neutralize free radicals via hydrogen atom transfer, while carotenoids quench singlet oxygen and scavenge peroxyl radicals. Additionally, due to different solubility, their physiological bioavailability and distribution to cell compartments differ significantly. Therefore, when combined, these compounds can exhibit synergistic effects, enhancing overall biological capacity beyond the sum of their individual actions. This is particularly relevant for obtaining functional extracts, as consumers increasingly seek multicomponent products that deliver broad-spectrum health benefits. In that context, extracts optimized for targeted functional characteristics are better positioned to meet both efficacy and market demand for multifunctional, natural health solutions. As such, the two following methods for the determination of antioxidant activity are frequently applied—antioxidant activity against the 2,2-azino-bis (3-ethylbenzothiazoline-6-sulfonic acid) (ABTS) radical, which reacts with both hydrophilic and lipophilic antioxidants, and activity against the 2,2-diphenyl-1-picrylhydrazyl (DPPH) radical, which predominantly reacts with lipophilic compounds [23].

While citrus peels supply approximately 85% of commercial pectin globally [24], mandarin remains understudied despite its potential. Emerging studies demonstrate mandarin peel pectin yields comparable to conventional sources: for example, Satsuma mandarin peel yields 15.34–18.99% pectin using high-hydrostatic-pressure-assisted methods [25]. The possibilities of the MAE of pectin from mandarin peel have not been thoroughly investigated. Karbuz and Tugrul [26] showed that MAE was superior to ultrasound-assisted extraction in terms of improved pectin yields and purity. Kumar and co-workers [27] applied MAE to compare grapefruit and mandarin peel as sources of pectin and investigated the impact of mandarin peel pretreatment on the yields and purity of the obtained pectin. Even though both investigations pointed out the advantages of MAE as an extraction technique and the applicability of mandarin peel as a source of high-quality pectin, again, as in the case of polyphenols, a kitchen microwave oven was applied for extraction. Consequently, the authors failed to conduct in-depth multiple-response optimization in order to investigate the significance of particular MAE conditions on the yields and characteristics of the obtained pectin.

Generally, current scientific research on the potential utilization of secondary raw materials, such as mandarin peel, primarily focuses on reducing the environmental footprint of developed procedures while improving the yields and quality of obtained products. Therefore, special emphasis is set on investigating solvent-free extraction or the utilization of green solvents and the application of contemporary extraction methods targeting those with a high level of technological readiness and potential for industrial application. Another innovative strategy is the development of a biorefinery approach that supports circular bioeconomy principles since it combines different biomass conversion processes to produce high-value-added products. It offers wide range of advantages in terms of a reduced environmental footprint (due to the focus on green processing technologies, improved resource efficiency and reduced waste generation), economic benefits (due to the consolidation of extraction steps, which reduces operational costs and infrastructure requirements, shorter extraction times or improved yields) and simplified scale-up (due to a reduced need for multiple separate processing lines) [28].

This research presents a comprehensive investigation into the application of MAE for isolating polyphenols, carotenoids and pectin from mandarin peel in the laboratory closed-vessel microwave extraction system that offers numerous benefits compared to a household microwave oven. In addition to aiming for maximal yields of major target compounds, it also investigates the possibility of obtaining the highest functionality (antioxidant activity) of the final product. Moreover, the focus is set on the application of green solvents—citric acid for pectin extraction and water–ethanol mixtures for the extraction of polyphenols and carotenoids. By utilizing multiple-response optimization, the study aims to elucidate the influence of specific MAE parameters on both the yield and quality of the extracted compounds. Furthermore, the work provides a comparative analysis of MAE versus CSE in terms of energy consumption, extraction efficiency and chemical composition of the products. Finally, the potential integration of optimized MAE processes into a continuous, two-step process is investigated. In this biorefinery approach, pectin is extracted from the residue remaining after the extraction of antioxidants, highlighting the numerous advantages in terms of enabling sustainable and efficient valorisation of mandarin peel.

## 2. Materials and Methods

### 2.1. Organization of Conducted Research

For antioxidant recovery, Satsuma mandarins (*Citrus unshiu* Marc.) were sourced from the local mandarin juice producer Novallis, located in Opuzen, Croatia. The fruits were manually peeled, and the peels were frozen at −50 °C and subsequently lyophilized for 36 h using an ALPHA freeze dryer (Christ, Osterode am Harz, Germany). Following lyophilization, the dried mandarin peels were milled to achieve a uniform particle size. The resulting material was then passed through a 0.45 mm stainless steel sieve to ensure homogeneity. The obtained powdered sample was stored at −20 °C until further experimentation. For pectin extraction, mandarin peel was dried in a laboratory oven at 60 °C until constant weight. After drying, the peel was ground, sieved through a 0.8 mm sieve and stored at 4 °C until extraction.

**Figure 1:**
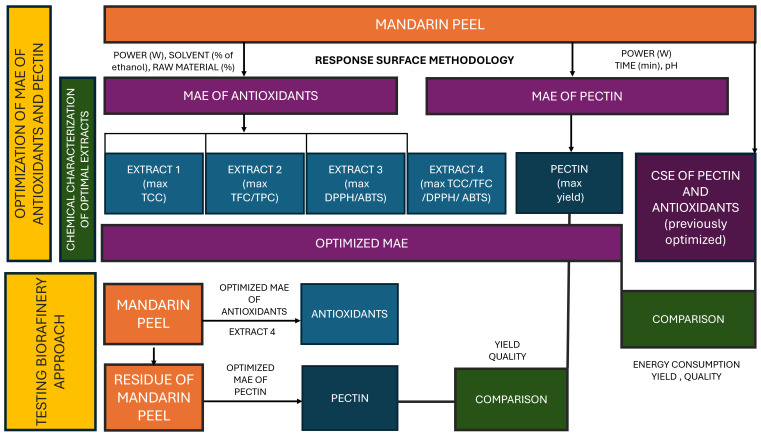
A schematic diagram of the experiments performed in this study. MAE—microwave-assisted extraction; TCC—total carotenoid content; TFC—total flavonoid content; TPC—total polyphenol content, ABTS—2,2-azino-bis (3-ethylbenzothiazoline-6-sulfonic acid) radical scavenging assay; DPPH—2,2-diphenyl-1-picrylhydrazyl radical scavenging assay.

A schematic diagram of the research performed in this study is presented in Figure 1.

Briefly, in the first phase of the investigation, response surface methodology (RSM) was used to optimize ethanol–water MAE of polyphenols and carotenoids form mandarin peel and determine major factors influencing yields. All together, 4 processes were optimized with the aim of obtaining extract 1 with maximal total carotenoid content; extract 2 with maximal content of polyphenols and flavonoids; extract 3 with maximal antioxidant activity and extract 4 where MAE conditions were optimized to yield maximal amounts of all targeted compounds. Parallelly, RSM was applied to optimize MAE for the extraction of pectin from mandarin peel. In order to investigate potential benefits of MAE, optimized MAE procedures were compared to optimal CSE procedures in terms of yields and energy consumption. For polyphenols, carotenoids and antioxidant potential, CSE procedures were optimized in the framework of preliminary investigations (unpublished data); for referent pectin, a CSE method available in the literature was used. In the second phase of the investigation, residues of mandarin peel remaining after obtaining extract 4 were used for the MAE of pectin. The characteristics of the obtained pectin were compared with those obtained from intact mandarin peel to investigate the applicability of the proposed biorefinery approach.

### 2.2. Experimental Design for Optimizing Extraction of Antioxidants

RSM coupled with the Box–Behnken design (three factors/three levels) (DesignExpert 7.0.0, Stat-Ease, Minneapolis, MN, USA) was used for the optimization of the MAE of antioxidants.

Prior to conducting a response surface analysis, initial factor screening using experimental designs such as Taguchi methods, Plackett–Burman designs, fractional factorial designs or single-factor-at-a-time experiments is critical to identify statistically significant parameters, thereby reducing experimental complexity and optimizing resource allocation by focusing subsequent surface exploration on influential variables [29,30,31]. In this case, preliminary research was conducted using a one-factor design, monitoring the influence of the solvent-to-raw material ratio; solvent type (ethanol, ethanol water mixtures or methanol) and temperature on the yields of polyphenols, flavonoids, carotenoids and antioxidative activity. Based on preliminary results, the following conditions were chosen for the RSM: ethanol–water mixtures as solvents (ranging from 30 to 90% *v*/*v*); microwave power (400–800 W) and the time of extraction adjusted to keep the maximal temperature of the reaction mixture below 75 °C to prevent the degradation of carotenoids. 

Independent factors and their levels are shown in Table 1, with the following parameters—raw material (0.1–0.3%, *w*/*v*), power (400–800 W) and ethanol/water solvent ratio (30–90%, *v*/*v*)—while the temperature was monitored and adjusted to be below 75 °C. In that context, extraction was performed for 4 min (at a power of 400 W), for 2 min (600 W), and for 1 min (800 W) in a laboratory closed-vessel microwave system (Ethos Easy, Milestone, Sorisole, Italy). After extraction, the reaction mixtures were cooled down to room temperature and filtered. The obtained filtrates were used for the determination of antioxidant capacity, total carotenoid, total polyphenolic and total flavonoid content. A total of four optimal extracts were obtained, depending on the desired outcomes: 4 optimizations were performed by either maximizing selected responses (extracts 1–3: total carotenoids, total polyhenols/flavonoids, antioxidant activity) or by maximizing all responses (extract 4).

CSE of antioxidants was optimized in the frameworks of preliminary investigations. The Box–Behnken design (three factors/three levels) was applied. Independent factors and their levels were as follows: raw material (0.1–0.3%, *w*/*v*), temperature (25–65 °C) and ethanol/water solvent ratio (30–90%, *v*/*v*). Optimization was equivalent to the optimization of MAE, and therefore, a total of 4 optimal extracts were obtained (extract 1–extract 4). Equations describing the relations between the CSE conditions and observed variables are presented in Table S1. Other data regarding CSE optimization are not shown in this study and are available on request.

### 2.3. Experimental Design for Determination of Pectin

RSM coupled with the Box–Behnken design (three factors/three levels) (Stat-Ease 360 22.0.8, Stat-Ease, Minneapolis, MN, USA) was used to optimize the MAE conditions for pectin. Independent factors and their levels are shown in Table 2.

MAE was performed using a laboratory closed-vessel microwave system (Ethos Easy, Milestone, Sorisole, Italy). Each extraction began with a 10 s phase to reach the set microwave power, followed by a constant power phase for the designated extraction time, and ended with a cooling phase until the temperature reached 85 °C. The stirring speed was set to 10%. Time and power were chosen as independent factors based on extensive literature data pointing them out as the mostly influential in MAE [32]. Citric acid has been chosen as the solvent due to its good ecological acceptance and the literature pointing it out as particularly suitable for the extraction of pectin from similar matrices (citrus fruit) [33]. In addition to power and time, temperature can also have significant impact on pectin yields. It was not considered in the experimental design since the majority microwave digestion units (including ours) do not have an integrated cooling system installed that would enable the regulation of the temperature of the reaction mixture at the constant microwave power.

Temperature change was monitored during the process. A total of 1 g of mandarin peel was mixed in the vessel with 20 mL of 1% citric acid monohydrate (with the pH adjusted to 1, 1.5 or 2 using 6 M hydrochloric acid). The sample-to-solvent ratio was chosen based on available literature data and conducted preliminary research. Vessels were then closed and placed in the microwave rotor plate. Afterwards, the extract was passed through cotton gauze directly into a sinter funnel (grade 2) with FN3 filter paper and vacuum filtered, followed by a second filtration with filter paper. To precipitate pectin, an amount of 96% ethanol equal to twice the volume of the extraction solvent was added to the filtrate and the mixture was refrigerated at 4 °C. After 24 h, pectin was collected by filtration, dried at 45 °C until constant weight, ground in a mortar and stored at 4 °C.

The CSE of pectin was performed as described by Casas-Orozco et al. [34], with some modifications. Briefly, 5 g of mandarin peel was extracted in a water bath (2 h/85 °C) with 100 mL of 1% citric acid monohydrate (with the pH adjusted to the optimal MAE pH). All procedures following extraction were the same as described for MAE.

Once the optimal MAE conditions for polyphenol and pectin extraction were determined, polyphenols were extracted from lyophilized mandarin peel. After filtration, the residue was collected, dried and used for pectin extraction. The pectin’s yield and characteristics were compared to pectin extracted from the sample where polyphenols were not extracted.

### 2.4. Determination and Chemical Characterization of Antioxidants

#### 2.4.1. Spectrophotometric Determination of Antioxidants

Total carotenoid content (TCC) in mandarin peel extracts was determined according to the modified Barros et al. [35] method. Detection was performed by measuring absorbance at 453, 505 and 663 nm using a UV-Vis spectrophotometer (Model SpectraMax i3x Multi-Mode Detection Platform, Molecular Devices, San Jose, CA, USA). The content was calculated using Equation (1), and results are presented as µg of beta-carotene equivalent per g of dry peel.

(1) β−carotene(mg/100 mL)=(0.216×A663)−(0.304×A505)+(0.452×A453)

Total phenolic content (TPC) was determined by the modified Ainsworth & Gillespie et al. [36] method using the Folin–Ciocalteu (F-C) reagent (obtained from Merck, Darmstadt, Germany). A volume of 20 µL of extract and 50 µL of 10% (*v*/*v*) F-C reagent was added to the microplate, shaken and incubated for 5 min at 37 °C. Furthermore, 160 µL of 700 mM Na_2_CO_3_ (Lach-Ner, Tovarni, Czech Republic) was added and incubated for 30 min at the same temperature. Absorbance was measured at 750 nm, using a UV-VIS spectrophotometer (Model Victor x3, PerkinElmer, Waltham, MA, USA). The results are expressed as mg of gallic acid equivalents (GAE) per g of dry peel.

Total flavonoid content (TFC) in mandarin peel was detected using the spectrophotometric method of Ivanova et al. [37]. A volume of 4 mL of water and 1 mL of mandarin peel extract was mixed with 0.3 mL of NaNO_2_ (0.5 g/L) in a volumetric flask. After 5 min, 0.3 mL of AlCl_3_ (Sigma Aldrich, Steinheim, Germany) (1 g/L) was added. The reaction mixture was left for 6 min to react when 2 mL of NaOH (Sigma Aldrich, Steinheim, Germany) was added (1 mol/L). The volume was adjusted up to 10 mL with distilled water. Measurements were performed by measuring the absorbance at 360 nm using a UV-Vis spectrophotometer (Model SpectraMax i3x Multi-Mode Detection Platform, Molecular Devices, San Jose, CA, USA). The content of total flavonoids was quantified, and the results are expressed as mg quercetin equivalents (QE) per g of dry peel.

For the determination of antioxidant activity, DPPH and ABTS reagents were used (obtained from Sigma Aldrich, Steinheim, Germany). Antioxidant capacity determination using ABTS reagent was modified according to Re et al. [38]. A volume of 20 μL of extract was pipetted in the microplate with 200 μL of diluted ABTS^∙+^ reagent. The absorbance was measured at 750 nm, using a UV-Vis spectrophotometer (Model SpectraMax i3x Multi-Mode Detection Platform, Molecular Devices, San Jose, CA, USA), and the results are expressed as the mmol Trolox equivalent (TE) per g of dry peel. The other method was modified according to Brand-Williams et al. [39]. In total, 150 μL of the DPPH methanol solution was mixed with 150 μL of extract. After 30 min of incubation, absorbance was measured at 530 nm, using a UV-VIS spectrophotometer (Model Victor x3, PerkinElmer, Waltham, MA, USA), and the results are expressed as mmol Trolox equivalent per g of dry peel. 

#### 2.4.2. HPLC Analysis of Polymethoxyflavones

In this research, optimal extracts were characterized by the determination of polymethoxyflavones (PMFs) using high-performance liquid chromatography, HPLC. All samples were evaporated to dryness and dissolved in 4 mL of water. A mixture was prepared by combining 0.6 mL of sample with 0.6 mL of a methanol/DMSO solution (1:1, *v*/*v*) in Eppendorf tubes. The mixture was then sonicated for 15 min at 50 °C, followed by centrifugation at 5000 rpm for 15 min at 4 °C. The resulting supernatant was transferred and filtered by 0.45 μm LLC-RC syringe filters prior to analysis to vials for HPLC analysis. A modified method was used to determine the content of nobiletin and tangeretin by HPLC in the obtained extracts [17]. Nobiletin and tangeretin were obtained from Toronto Research Chemicals (Toronto, ON, Canada).

The Agilent 1260 Infinity II System (Agilent, Waldbronn, Germany), equipped with an autosampler, column thermostat and diode-array detector (DAD), was used. The separation of nobiletin and tangeretin was performed on an Agilent Poroshell 120 SB-C18 column (4.6 × 150 mm, 4 μm) at 40 °C. The detection wavelengths were 280, 330 and 360 nm. The injected sample volume was 20 μL. The mobile phase was 2% formic acid (solvent A) and methanol (solvent B) at a flow rate of 0.8 mL/min. The solvent gradient in volume ratios was as follows: 0–10 min, 10–20% B; 10–20 min, 20–30% B; 20–30 min, 30–40% B; 30–35 min, 40% B; 35–42 min, 40–50% B; 42–52 min, 50–90% B; 52–53 min, 90–10% B; and 53–60 min, 10% B. Polymethoxyflavones were identified by matching the retention time of each chromatographic peak with external standards and the DAD spectrum. The quantification of individual PMF peaks was performed by the external standard method. Results are expressed as mg per g of dried mandarin peel. HPLC chromatograms are presented in Figure S1.

### 2.5. Characterization of Pectin

The physico-chemical characterization of pectin included the determination of yield, equivalent mass (EM), methoxyl content (MC), the degree of esterification (DE) and anhydrouronic acid content (AUA), as described in previous publications [40], along with Fourier transform infrared (FTIR) spectroscopy for functional group identification. All titrations were performed using standardized 0.01 M NaOH.

The pectin yield from both CSE and MAE was determined gravimetrically using Equation (2):

(2) $$Pectin\;yield\left(\%\right)=\frac{w(pectin)}{w(mandarin\;peel)}\times 100$$

where w is dry weight in grams.

For the determination of EM, the pectin sample (0.05 g) was moistened with 200 µL of 96% ethanol, dissolved in 10 mL of carbon dioxide-free water and left closed overnight in a magnetic stirrer to allow complete pectin dissolution. After the addition of 100 mg of NaCl and 1 drop of phenol red indicator, the mixture was titrated until the pink endpoint was reached. EM was calculated using Equation (3):

(3) $$EM\left(g/mol\right)=\frac{Weight\;of\;sample\left(g\right)\times 1000}{Volume\;of\;alkali\left(mL\right)\times Molarity\;of\;alkali}$$

This neutralized solution was used for the determination of MC, by adding 2.5 mL of 0.25 M NaOH and leaving the mixture closed at room temperature. After 30 min, 2.5 mL 0.25 M HCl was added and titrated to the pink endpoint. MC was calculated from Equation (4):

(4) $$MC\left(\%\right)=\frac{Volume\;of\;alkali\;\left(mL\right)\times Molarity\;of\;alkali\times 31}{Weight\;of\;sample(g)\times 1000}\times 100$$

where 31 is the molecular weight of the methoxyl group.

For the determination of the DE, the pectin sample (0.05 g) was moistened with 500 µL of 96% ethanol, dissolved in 5 mL of distilled water and left closed overnight in a magnetic stirrer to allow complete pectin dissolution. After adding 1 drop of phenolphthalein, the solution was titrated until the pink endpoint was reached (initial titration volume). To neutralize polygalacturonic acid, 2.5 mL of 0.1 M NaOH was added, and the sample was vortexed for 30 s and left at room temperature for 2 h to allow pectin deesterification. Then, 2.5 mL of 0.1 M HCl was added along with 1 drop of phenolphthalein, and the mixture was titrated again until the pink endpoint was reached (final volume). The DE was calculated from Equation (5):

(5) $$DE\left(\%\right)=\frac{Final\;titration\;volume\;(mL)}{Initial\;titration\;volume\left(mL\right)+Final\;titration\;volume\left(mL\right)}\times 100$$

AUA was calculated from the equation below using titration volumes obtained from the determination of EM and MC by Equation (6):

(6) $$AUA\;(\%)=\frac{176\times 0.01z\times 100}{w\times 1000}+\frac{176\times 0.01y\times 100}{w\times 1000}$$

where a molecular unit of AUA (1 unit) = 176 g, z = mL (titre) of standardized 0.01 M NaOH used in the determination of EM, y = mL (titre) of standardized 0.01 M NaOH used in the determination of MC and w = weight of the sample in grams.

The obtained pectin was analyzed using a FTIR spectrophotometer (Spectrum Two, Perkin Elmer, Waltham, MA, USA), over a spectral range of 500–4000 cm^−1^ with 16 scans and a spectral resolution of 4 cm^−1^.

### 2.6. Energy Consumption

Total energy consumption for the optimized CSE and MAE protocols was calculated according to Equation (7):

(7) $$E(kWh/kg)=\frac{P(kW)\times t(h)}{K(kg)}$$

where P is the power of the water bath/microwave extraction unit (kW); t is the time of heating/microwave radiation during extraction duration (h) and K is the capacity of the water bath/microwave extraction unit (kg), referring to the maximal mass of raw material that can be processed in one run due to the size/design of the water bath/microwave unit. The time of heating for CSE was the sum of the time needed for the water bath to heat to the targeted extraction temperature and the time the heater was working during the extraction process. In MAE, the instrument was at 100% power during the whole time of extraction. For CSE, the power of the water bath was 1.5 kW; K was 0.09 kg for pectin and 0.0018–0.0054 kg for antioxidants (depending on the conditions of the extraction procedure). For MAE, the power for pectin and antioxidants was 1.335 kW and 0.4 kW, respectively, while K was 0.0375 kg and 0.0008–0.0012 kg, respectively. Energy consumption for the CSE, calculated using Equation (7), was additionally corrected with the energy needed for the shaking of the water bath, approximated to 0.1 kW/h (data obtained from the producer).

### 2.7. Statistical Analysis

Design-expert 7.0.0 and Stat Ease 360 22.0.8 software were used for the RSM. GraphPad Prism 10.4.1 (GraphPad Software, San Diego, CA, USA) was used for conducting other statistical tests and for the graphical presentation of data. ANOVA and Student’s *t*-test have been used for the calculation of significant differences between the obtained results. Analyses were conducted in 2–6 parallels (depending on the method) and obtained values were presented as means ± standard deviations.

## 3. Results

### 3.1. Optimization of Microwave-Assisted Extraction of Antioxidants

Response surface methodology was used to optimize the extraction parameters and obtain a model describing the yield of carotenoids, polyphenols, flavonoids and antioxidative activity (ABTS and DPPH) influenced by three independent variables. Single-factor and Box–Behnken designs were used for extraction optimization. Firstly, the effects of important aspects of the MAE process, like raw material, solvent ratio, power, time and temperature, on the yield of desired compounds were investigated in the single-factor experiment. Secondly, the Box–Behnken design was used to optimize the extraction process consisting of raw material (0.1–0.3%, *w*/*v*), ethanol/water solvent ratio (30–90%, *v*/*v*) and power (400–800 W). The experiment had 17 experimental runs, which were carried out in random order. Analysis of variance was used to assess the credibility of the model, and adequacy was assessed using the following criteria—*p*-value R2 (coefficient of determination), adjusted-R2, the coefficient of variation (CV), a lack of fit and adequate precision [41]—all of which are presented in Table S3. *p*-values were used to test the statistical significance of each term in the model. The five response variables presented in Table 3 are the means of the measurements (n = 5).

*p* < 0.0001 implies the model for TCC is significant. Conversely, the lack of fit (*p* = 0.1698) was not significant. The signal-to-noise ratio is measured by “Adeq Precision”, and a ratio greater than 4 is desirable [41]. The ratio of 40.87 indicates an adequate signal. A high value of R2 (0.9970) shows that the model successfully accounted for 99% of the variance of the observed dependent variable. The Adj R-Squared value (0.9931) indicates a high correlation between predicted and observed values, and the same indicates that the model can be applied to future data.

**Figure 2:**
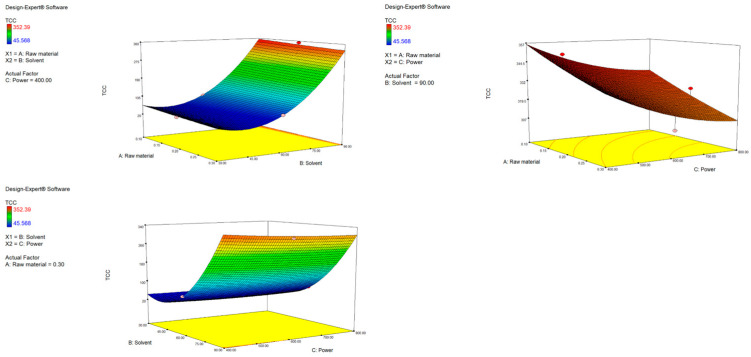
Response surface plots (3D) for TCC as a function of power, raw material and solvent.

The second-order polynomial equation for TCC is presented in Table S2. It demonstrates that the yield of carotenoids is influenced by both first- and second-order terms. As shown, factors A (raw material) and C (power) have a negative impact on TCC, while factor B (solvent) has a positive one. A similar trend can be seen when analyzing the *p* values of the terms in the equation. Significant influence is assigned to factor A (*p* = 0.0001), B (*p* < 0.0001) and the quadratic term B2 (*p* < 0.0001). Interestingly, the quadratic nature of the solvent variable that follows a U-shaped curve means it reduces the TCC yield to a certain point when its influence is opposite and nonlinear, meaning that slight changes in the variable lead to a fast increase in TCC. This was expected, since ethanol is a less polar solvent compared to water and can be more effective for carotenoid extraction. Simply put, increasing the solvent ratio increases TCC, while an increase in power and raw material decreases TCC. This is visually represented by response surface plots (Figure 2). The highest yield was obtained with the highest ethanol ratio (90%). The interactions among independent variables were not shown to be significant; however, response surface plots do reveal some important trends. With the highest used ethanol ratio (90%) as a fixed parameter, increasing power up to 800 W resulted in a decrease in TCC, probably resulting in carotenoid degradation under strong microwave radiation [42]. When keeping the raw material fixed, it is obvious that the more ethanol, the more efficient the extraction, independent of the power used. As presented in Table 3 the obtained values of TCC ranged depending on the applied extraction conditions, from 45.57 to 352.39 µg β-carotene/g. Similar results were found in the research by Maslov Bandić et al., where TCC from mandarin peel ranged from 58.67 to 227.18 µg β-carotene/g [43]. Comparable yields were also obtained from citrus and orange peel by other authors [44,45].

For TPC extraction, a quadratic model was suggested (*p* = 0.0104). The lack of fit (*p* = 0.0304) was statistically significant; on the other hand, the residual plot was characterized by random scattering and constant variance (Figure S3B). A ratio of 11.064 indicates an adequate signal. The coefficient of variation (CV) is 5.86. In order to verify the model’s repeatability, a CV of less than 10 is desirable, according to Beg et al. [46]. Almost 90% of the variation can be explained by the projected model proved by R2 0.8949. An Adj R-Squared value of 0.7599 indicates a good correlation between predicted and observed values. In the case of TFC, the suggested quadratic model is statistically significant (*p* = 0.0042). The lack of fit is not significant (*p* = 0.2790), and the signal is adequate, indicated by the ratio of 9.858. A CV of 4.14 is satisfactory. The model describes the relationship between the response and independent variables well, as can be seen from the high values of R2 (0.9207) and adj-R2 (0.8187) [47]. The final equation in terms of coded factors for TPC and TFC is presented in Table S2.

The model showed that all individual terms had a statistically significant negative effect: A—raw material (*p* = 0.0210), B—solvent (*p* = 0.0139) and C—microwave power (*p* = 0.0475). Furthermore, the BC interaction had a significant negative effect (*p* = 0.0252). Term B2 also had a significant impact on the yield (*p* = 0.0018), where the term has a (-) sign, meaning it follows a reverse U-shaped curve. The increase in ethanol concentration leads to an increase in TPC up to a certain level, when that effect becomes reversed. The same was noticed in the case of TCC but in the opposite direction, which is logical when considering the different polarity of polyphenols and carotenoids [48].

**Figure 3:**
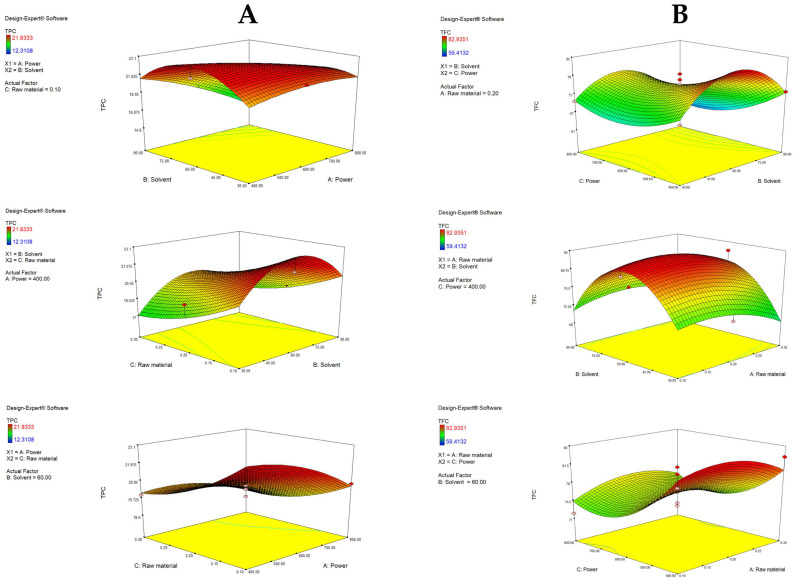
Response surface plots (3D) for TPC (**A**) and TFC (**B**) as a function of power, raw material and solvent.

Figure 3A visually describes the effects of independent variables and their interactions on TPC. With fixed raw material, an increase in ethanol ratio from 30 to 60% increased the TPC. Also, under fixed power, a lower raw material ratio (0.1%) resulted in a higher TPC (22.36 mg GAE/g d.w.), indicating that more solvent (compared to raw material) is needed to make the extraction of polyphenols efficient. As previously mentioned, it was visually confirmed that higher ethanol ratios significantly decrease the TPC yield (to 16.58 mg GAE/g). As seen in the third graph, with a fixed solvent, a decrease in power increases TPC. Shorter exposure intervals at low-to-moderate power may preserve or even increase TPC due to more suitable extraction conditions. On the other hand, longer exposure times at higher microwave power cause thermal degradation of phenolic compounds [49,50]. This model has shown that the highest yield of polyphenols can be obtained using 60% ethanol, with low power and a low amount of raw material. Similar findings were reported by García-Martín et al. [51], which determined the highest TPC with 50% ethanol.

When analyzing TFC, C (power) showed a statistically significant impact (*p* = 0.0174), specifically C2 (*p* = 0.0166), as well as solvent B2 (0.0003). The solvent showed a similar trend as described for TPC. Interestingly, the power had a more pronounced effect for TFC, where an increase from 600 to 800 W increased TFC slightly, while an increase in power from 400 to 600 W resulted in a decrease, clearly following the U-shaped curve of the quadratic term.

Figure 3B graphically demonstrates the effects of the tested variables on TFC. A rise in the yield of flavonoids was obtained with an increase in solvent up to 60%, while a further increase in the ethanol ratio resulted in a decrease in TFC. Additionally, more raw material, up to a central point (0.2%), resulted in a higher yield, while further addition of raw material resulted in a decrease. The highest TFC was determined with the lowest power (400 W), again emphasizing the sensitive chemical nature of flavonoids. With fixed power, raw material and solvent did not significantly influence TFC. Overall, TPC in the extracts ranged from 12.31 to 21.83 mg GAE/g, while TFC ranged from 59.41 to 82.94 mg QE/g, which is comparable to the results of other authors. Zhang et al. [23] determined TPC to be in the range from 22.80 to 32.76 mg GAE/g d.w., while Kumar et al. [52] quantified it at 22.88 to 43.61 mg GAE/g d.w. in Citrus reticulata peels. Furthermore, Maslov Bandić et al. [43] determined TPC in the range from 22.24 mg GAE/g to 28.77 mg GAE/g. On the other hand, the same authors obtained TFC in a range from 25.87 mg QE/g to 35.90 mg QE/g, depending on the mandarin variety. Similarly, Lee & Kim [53] found TFC in the range from 23.51 to 43.99 mg QE/g in mandarin peels of different maturation stages.

Furthermore, the impact of extraction conditions on ABTS antiradical activity was described using a quadratic model (*p* = 0.0001). The Lack of fit (*p* = 0.1576) was not significant, and the adequate precision was greater than 4 (16.953), which indicates an appropriate signal. R2 was 0.9671, with Adj R2 at 0.9249, confirming that the model successfully described the majority of variation in the dependent variable. The quadratic model for DPPH was significant (*p* = 0.0047), and the lack of fit (*p* = 0.0041) was statistically significant; however, the residual plot illustrates random scattering with no observable trends and constant variance (Figure S3D). A ratio of 10.457 indicates an adequate signal. Almost 92% of the variation could be explained by the projected model, according to the R2 value of 0.9181. An Adj R-Squared value of 0.8128 indicates a good correlation between predicted and observed values. The coefficients of variation (CVs) were satisfactory: 5.12 (ABTS) and 5.61 (DPPH). The final equations in terms of coded factors for antioxidative activity are shown in Table S2.

**Figure 4:**
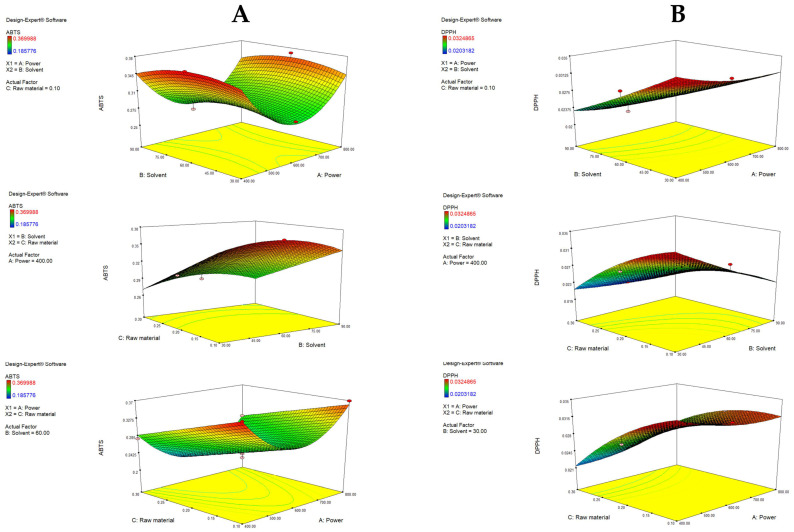
Response surface plots (3D) for ABTS (**A**) and DPPH (**B**) as a function of power, raw material and solvent.

It can be observed that the increase in raw material significantly (*p* = 0.0001) decreases ABTS antiradical activity. Furthermore, the solvent follows a quadratic function (B), similar to the case of TFC. The term power also follows a quadratic function (C2). Figure 4A depicts response surface plots, and it can be seen that an increase in radical inhibition was achieved with an increase in ethanol from 30 to 60% (when the raw material is fixed), while a further increase resulted in the lowest antioxidant activity. This indicates that there is a central area that provides the most efficient extraction of antioxidants, probably due to the simultaneous extraction of both polar and less polar antioxidants [48]. The power increases to the middle point (600 W) resulted in a decrease in the antioxidative potential, while a further increase had a positive effect. With fixed power, a lower amount of raw material increases ABTS, and a higher ethanol ratio increases the antioxidative potential due to the greater capacity of the solvent to extract the antioxidants if it is present in a high ratio compared to the raw material. In that context, the highest ABTS values can be observed with the lowest power, the lowest raw material amount and 50% ethanol.

DPPH was significantly affected by the A—raw material (*p* = 0.0042), A2 (*p* = 0.0353), B—solvent (*p* = 0.0016) and C—power (*p* = 0.0480), along with the interaction of raw material and solvent (*p* = 0.0122) and raw material and power (*p* = 0.0248). Visualized in Figure 4B, the optimal raw material percentage for maximizing DPPH was 0.1%, and further increases had a negative impact on the yield. An increase in the ethanol ratio also decreased the DPPH antioxidative effect. Higher antioxidative activity was noted when power was increased, which is consistent with the results of Hayat et al. [14]. The interaction between raw material and solvent, as well as raw material and power, showed significant impact, meaning that if an increase in raw material occurs along with an increase in the ethanol ratio or power, a considerable amount of antioxidants can be extracted, which results in notable activity.

Antioxidant activity ranged from 0.19 to 0.37 mmol TE/g and 0.02 to 0.03 mmol TE/g for ABTS and DPPH antiradical activity, respectively. The obtained results are comparable to the values obtained by other authors, although there is a high level of variation. This is probably due to variable extraction conditions and differences in mandarin genotypes, agroclimatic conditions and sample handling [23]. Despite the high variability, all available literature data show ABTS antioxidant activity to be significantly higher compared to DPPH, which is consistent with our results [21,54,55]. The abovementioned observations can be explained by the two radicals’ different mechanisms of reaction [56,57]. DPPH radical reacts predominately with antioxidants with lipophilic characteristics (such as TCC), while ABTS reacts with both lipophilic and hydrophilic antioxidants (TPC, TFC and TCC) [23]. In the analyzed extracts, TCC was relatively low in relation to TPC and TFC, resulting in lower DPPH antiradical activity.

The optimal conditions obtained using these models were as follows: extract 1, in which TCC was maximized (raw material—0.11%, power—400 W, ethanol ratio—90%), extract 2, with the maximization of TPC and TFC (raw material—0.14%, power—400 W, ethanol ratio—54%), extract 3with maximized antioxidative activity (raw material—0.10%, power—400 W, ethanol ratio—41%), and extract 4, where all the responses were maximized (raw material—0.16%, power—400 W, ethanol ratio—77%). The temperature of extraction was always kept below 75 °C because flavonoids have been shown to be heat-sensitive [58]. Also, the oxidative degradation, free radical production and isomerization of carotenoids are influenced by extraction time and temperature [59].

#### Optimization and Validation of the Predicted Model

Experimental validation was performed to compare the predicted and experimentally obtained results. In that context, TCC values were in the range of 352.267 ± 17.402 µg β-carotene/g, which is comparable to the predicted ones (354.064 µg β-carotene/g) in optimal extract 1. In the case of TPC and TFC, the values were 21.764 ± 0.456 mg GAE/g and 83.128 ± 1.979 mg QE/g, compared to the predicted values according to the model (TFC—83.283 mg QE/g and TPC—21.878 mg GAE/g) for optimal extract 2. Values of ABTS and DPPH in optimal extract 3 were 0.366 ± 0.016 and 0.033 ± 0.001 mmol TE/g compared to the predicted values 0.367 and 0.032 mmol TE/g. For optimal extract 4, where all the input parameters were maximized, the experimentally obtained results were TCC—189.230 ± 16.498 µg β-carotene/g, TFC—77.050 ± 1.557 mg QE/g, TPC—21.230 ± 0.993 mg GAE/g, DPPH—0.026 ± 0.001 mmol TE/g and ABTS—0.244 ± 0.010 mmol TE/g, compared to the predicted ones of 201.245 µg β-carotene/g, 80.191 mg QE/g, 20.803 mg GAE/g, 0.025 mmol TE/g and 0.335 mmol TE/g, respectively. As shown, the adequacy of the model used was confirmed, with a good correlation between the predicted and experimentally obtained results.

The desirability function (DF), which provides a desirability grade, was used to assess an optimization technique based on the Harrington scale. The results in Table 4 indicate that the desirability in the case of optimal extract 1 and 2 was 1. The findings indicated that improvement beyond this point has no preference [60]. The quality evaluation of extract 3 is acceptable and excellent (0.991), and for extract 4, the desirability is 0.711, which is in the range of 0.80–0.63, evaluated as acceptable but good.

To ensure that the results were reliable under optimal circumstances, validation tests were conducted. The model’s consistency and dependability were demonstrated by the relative standard error (n = 5) between the experimental and projected values, which was less than 10% [61] and is presented in Table 4.

**Figure 5:**
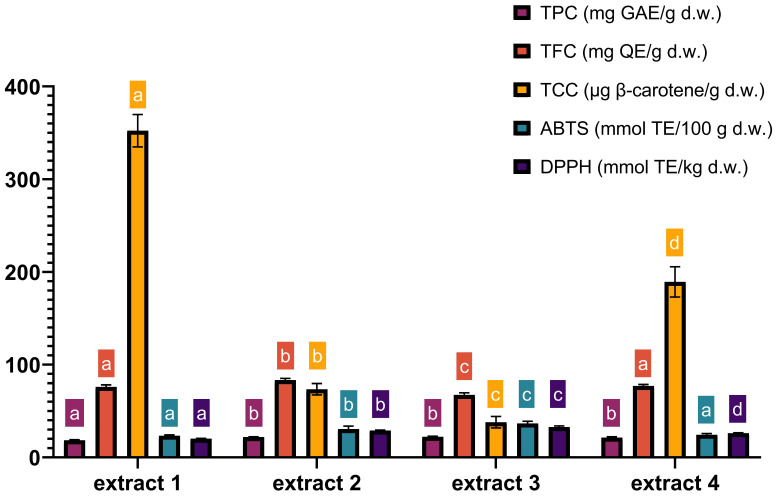
Characterization of TPC, TFC, TCC, ABTS and DPPH in four optimal extracts. TPC—total phenolic content; TFC—total flavonoid content; TCC—total carotenoid content; extract 1—maximized TCC (0.11% raw material/400 W power/90% ethanol); extract 2—maximized TPC and TFC (0.14% raw material/400 W power/54% ethanol); extract 3—maximized antioxidative activity (0.10% raw material/400 W power/41% ethanol); extract 4—maximized all responses (0.16% raw material/400 W power/77% ethanol). Time of MAE in all optimal extracts was 4 min. Columns marked with different letters belong to different statistical groups (*p* < 0.05). Columns and their corresponding statistical letters are colour-coded according to each antioxidant test.

The chemical composition of extracts 1–4, obtained under optimized MAE conditions, is presented in Figure 5. The data show that the most observable effect of extraction variables is the effect of ethanol concentration on TCC yields. In extract 4, which maximized the yields of all antioxidants, the content of TCC was 1.8 times lower compared to the values obtained with extract 1 (189.2 µg β-carotene/g and 352.27 µg β-carotene/g, respectively). Furthermore, ABTS and DPPH values were the lowest in extract 1 compared to the other optimal extracts, which was related to the highest percentage of ethanol. The content of nobiletin and tangeretin in extracts 1–4 obtained under optimized MAE conditions is presented in Table 5—as mentioned previously, their concentration was determined as they are as the most abundant among citrus flavonoids and have proven health benefits.

Nobiletin was quantified in a range from 0.57 to 0.70 mg/g while the content of tangeretin was lower, ranging from 0.13 to 0.14 mg/g. The obtained results are similar to the results of Zhang et al. [62], who obtained 0.51–6.49 mg/g nobiletin and tangeretin in a range from 0.19 to 2.92 mg/g d.w. in different mandarin genotypes. Another study by Chen et al. [63] also showed similar results for nobiletin, at 0.39–7.79 mg/g, while tangeretin was determined in lower concentrations, at 0.15 to 3.37 mg/g. Table 5 shows that tangeretin yields were not significantly affected by different MAE conditions. On the other hand, nobiletin was significantly influenced by MAE conditions, and the highest yields were obtained in extracts 1 and 4 (and, as expected, not in extract 2, which contained the highest TFC). This shows that, due to variability in the chemical characteristics of flavonoids, maximizing total flavonoid content does not necessarily maximize the content of particular flavonoid compounds.

### 3.2. Microwave-Assisted Extraction of Pectin

Response surface methodology with Box–Behnken design was employed to optimize the MAE of pectin from mandarin peel. Independent variables were extraction time, microwave power and pH, while response variables pectin were yield, AUA and DE. The model that best fit the data for yield was higher-order quadratic (*p* = 0.0167, R^2^ = 0.8780, adjusted R^2^ = 0.7211). The model showed that reaction time was a key variable influencing pectin yield (A-time, *p* = 0.0176), but it also recognized the significance of the interaction between time and power (AB, *p* = 0.0349), indicating that reaction time and power modulate this influence and should be mutually considered when optimizing the yield of the extraction. Microwave irradiation within a limited timeframe can lead to increase in the yield; however, prolonged irradiation can lead to its deterioration. The statistical significance of the quadratic term (A^2^, *p* = 0.0039) further shows that an increase in extraction time positively influences pectin yield to a specific point, after which that influence changes and starts to negatively impact yield.

AUA was described by a linear model (*p* = 0.031, R^2^ = 0.4828, adjusted R^2^ = 0.3635). pH (*p* = 0.0081) had a significant effect on the response variable. An acidic environment increases ester group hydrolysis, while keeping the AUA chains of the fibre stable, which may result in overall higher levels of AUA in pectin [64]. A similar phenomenon was observed for the DE (described by linear model (*p* = 0.0414, R^2^ = 0.4577, adjusted R^2^ = 0.3326), where pH also exhibited significant influence on the response variable. It is interesting to note that, although pH did not have a significant influence on yield in terms of statistical analysis of variance, it strongly affected the chemical characteristics of pectin. Model equations and additional statistical analyses can be found in the Supplementary Materials.

There were 17 runs in the optimization process (Table 6). The yield ranged from 4.19 ± 0.17% to 25.16 ± 1.64%. It can be seen in Figure S2 that the conditions for the optimal yield are in the central part of the surface area. The plots also reveal additional interactions between the independent variables, which might remain unaccounted for if the focus is put solely on the model statistics. A lower pH can improve the yield if the extraction time is short (under constant optimal conditions of power). Under these conditions, the extraction time is not sufficient for the release of pectin molecules and additional acidification of the environment can compensate for that. The interaction between pH and power (under the optimal value for time) is shown in Figure S2.

**Figure 6:**
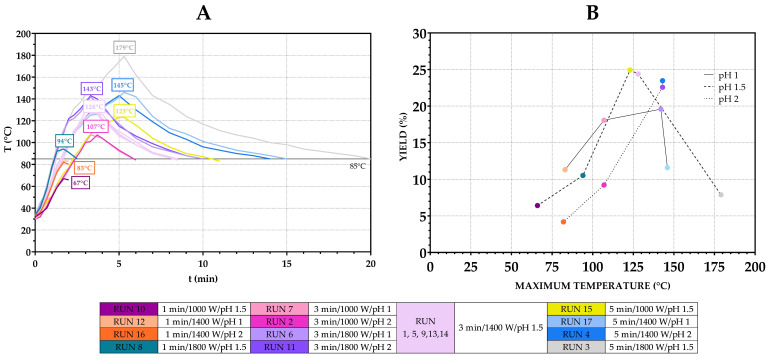
Impact of temperature on pectin yields. Temperature curves during microwave extractions (**A**) and yields achieved at maximum temperatures during runs (**B**).

An important variable in the extraction that was not included in the experimental design is the temperature (due to equipment shortages and the inability to precisely control both microwave power and temperature, as explained previously). It was monitored during extraction to evaluate its influence (alone and in interactions with other variables) on the pectin yield (Figure 6A). The relationship between the maximum achieved temperature, pH and yield is shown in Figure 6B.

Generally, at temperatures below 110 °C, the yield increased as the temperature increased. When comparing the yield from runs under identical power and time settings, it can be seen that acidic conditions may facilitate pectin extraction under mild thermal inputs: a higher yield was achieved with pH 1 compared to pH 2, at 11.32% vs. 4.19% (Runs 12 and 16) and 18.07% vs. 9.23% (Runs 7 and 2). This is in accordance with the interactions of pH and power/time as discussed previously (Figure S2). As temperature increased further, overall yield improved, reaching maximum values (above 20%) within an optimal temperature interval of 123–147 °C. In this range, the effect of pH reversed—a higher pH (pH 2) produced similar or better results compared to pH 1, 22.57% vs. 19.57% (Runs 11 and 6) and 23.47% vs. 11.57% (Runs 4 and 17), suggesting that a more acidic environment along with higher temperatures may accelerate degradation processes. Beyond this range, a further increase in temperature did not result in additional yield improvements, and there was noticeable decline in yield, likely due to thermal degradation. This was further supported by a visible colour change in pectin, obtained after Runs 3, 6 and 17, from light yellow-orange to darker orange/brown. Thus, while pH alone may not directly influence yield, its effect becomes pronounced in combination with heat, power and time, acting either as a facilitator of extraction or as a catalyst for degradation at elevated temperatures.

AUA was in the range of 55.7 ± 0.05 to 78.84 ± 0.74, as seen from Table 6, and DE from 38.20 ± 0.38 to 68.39 ± 2.45. Multiple-response optimization was performed on the following criteria: maximal yield (acceptable minimal value of 20% and maximal value of 30%), AUA in the range of 65–75% and a DE in the range of 42 to 62%. The optimal conditions for the extraction were 3.67 min, 1335 W and pH 1.49. The predicted and experimental results for the conformation run are represented in Table 7. It can be seen that the error in the prediction of AUA and yield was 10% or less, indicating satisfactory predictive potential of the model. The difference between the predicted and observed values was the highest for the DE, which is in accordance with the percentage of variability (R^2^) that could be described by the model as stated above in the text. Additional statistical analysis can be found in Table S4.

Previous studies utilized surface response methodology to optimize the MAE orange peel-derived pectin. Prakash Maran et al. [47] applied the Box–Behnken design to model the influence of microwave power, time, pH and solid–liquid on pectin yield. They achieved a considerably lower yield compared to our results, with the optimal conditions (422 W, 169 s, pH 1.4, solid: liquid ratio = 1:69 g/mL) resulting in a 19.24% yield. The optimal microwave power was markedly lower compared to ours, which could be the reason for the reduction in the yield. Also, the protocol in their study included washing the pectin with ethanol to obtain a pure pectin fraction, which probably resulted in certain losses in the process. Hosseini et al. [65] utilized sour orange peel as a source of pectin and optimized MAE using the Box–Behnken design. They reported very high R^2^ values (>0.9) for their models that explain the influence of time, power and pH on pectin yield and DE.

Similarly, Iñiguez-Moreno et al. [66] modelled the MAE of pectin using the central composite design. They tested the effects of time and temperature on extraction while keeping the microwave power at a constant value of 900 W. The optimal conditions were 4.9 min and 160.5 °C, resulting in 36.69% yield. These optimal conditions can be compared with those obtained in our study—the extraction time was higher compared to ours, due to the lower value of microwave power (900 W). The pectin obtained in their study had a DE above 50% in all tested conditions, while in our study, the DE ranged from a high- to lower-esterified form. Interestingly, they used a quadratic model to describe the influence of tested variables on the DE, while in our case, a linear model (pH having the main effect) was optimal to describe the behaviour of the mentioned variables. However, Iñiguez-Moreno et al. [66] did not include pH in their experimental design.

### 3.3. Comparison of MAE and CSE

#### 3.3.1. Comparison of MAE and CSE for Antioxidant Extraction

As mentioned previously, MAE has generally been demonstrated to improve compound recovery in a shorter time with a lower volume of solvent and usually reduced extraction times. Table 8 compares the yields of target antioxidants in extract 4 obtained by optimized MAE (0.16% raw material, 77% ethanol and power of 400 W) and CSE (0.13% raw material, 80% ethanol and 50 °C).

As shown, results obtained by both methods were comparable. Small, but statistically significant differences were obtained for polyphenolic compounds, while TCC was not influenced by extraction technique. Somewhat higher yields of TPP, TFC, DPPH, nobiletin and tangeretin were obtained by CSE while ABTS was significantly higher in MAE.

As can be observed from Table 9, the preparation of extracts using CSE requires from 5.0 to 16.8 times more energy when compared to the MAE (except in extract 3 where CSE is performed at room temperature). Furthermore, the CSE process was significantly longer.

#### 3.3.2. Comparison of MAE and CSE for Pectin Extraction

**Figure 7:**
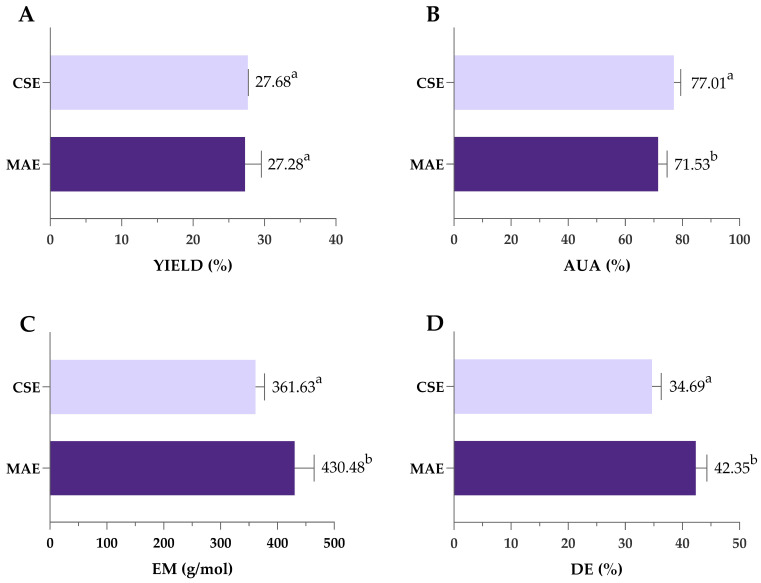
Comparison of yield (**A**), AUA (**B**), EM (**C**) and DE (**D**) of pectin obtained by optimized MAE and CSE. MAE—microwave-assisted extraction; CSE—conventional solvent extraction; AUA—anhydrouronic acid content; EM—equivalent mass; DE—degree of esterification. Columns marked with different letters belong to different statistical groups (*p* < 0.05).

Pectin obtained under optimal MAE conditions (3.67 min/1335 W/pH 1.49) was compared to pectin extracted by previously optimized CSE (120 min/85 °C/pH 1.49), as shown in Figure 7. Despite the significantly shorter extraction time, MAE achieved yields comparable to SLE (27.7 vs. 27.3%), highlighting its efficiency and potential as a rapid and effective alternative for pectin extraction. During MAE, the temperature peaked at 135 °C, in contrast to the constant 85 °C used in CSE. Higher microwave power and rapid heating contributed to efficient cell wall breakdown and high pectin yield achieved in a significantly shorter extraction time. This can be attributed to the mechanisms of microwave heating, which involve ionic conduction and dipole rotation. These processes rapidly generate heat within the solvent–sample matrix, promoting cell wall rupture and increasing the interaction between the solvent and plant material [67]. Previous studies have shown that microwave energy can cause the destruction of parenchymal cells, alter surface properties and inactivate enzymes that degrade pectin, all of which contribute to improved extraction efficiency [68].

Similar investigations that compared MAE and CSE for mandarin peel pectin extractions are scarce. The only available research on mandarin has been conducted by Kumar and co-workers [27] using the household microwave oven at 900 W (and not the closed-vessel system). In this work, both CSE and MAE resulted in significantly lower yields compared to our results, and MAE applied for 60 min showed yields comparable to CSE (18.58 vs. 14.85%). Investigations conducted on other types of citrus fruits are also partially in accordance with our results. Yeoh and co-workers [69] compared CSE (Soxhlet) and MAE for orange peel pectin extraction and obtained similar yields regardless of the applied extraction techniques. Rodsamran and Sothornvit [70] reported that MAE of lime peel at 700 W for 3 min, using citric acid and a 1:20 peel-to-extractant ratio, resulted in lower pectin yield compared to conventional heating. Unlike the mentioned studies, the present work used significantly higher microwave power (1335 W), which likely enhanced cell wall disruption and improved pectin release, resulting in fast and efficient extraction, which might explain observed differences. These findings suggest that microwave power plays a crucial role in optimizing extraction efficiency, which is in accordance with the results of our multiple-response optimization. However, higher pectin yields in this study might be due to the fact that we presented yields of raw pectin, which was not additionally purified after extraction (so that we could compare the impact of the extraction process on the purity of the obtained pectin). In previously mentioned research conducted by other authors, there are no specific data about conducting/omitting the purification step, so we cannot be sure if the yields reported refer to raw or purified pectin.

Pectin purity was also affected by the type of extraction—as shown in Figure 4B, both values of AUA exceeded the 65% threshold typically required for commercial-grade pectin, indicating high-quality extracts from both methods. However, AUA was significantly higher in pectin extracted by CSE (77.01%) compared to MAE (71.53%). Also, values of EM and the DE were significantly higher in pectin obtained by MAE; however, since all obtained values were lower than 50%, obtained pectin can be classified as low-methoxyl pectin regardless of the extraction type (Figure 7C,D).

**Figure 8:**
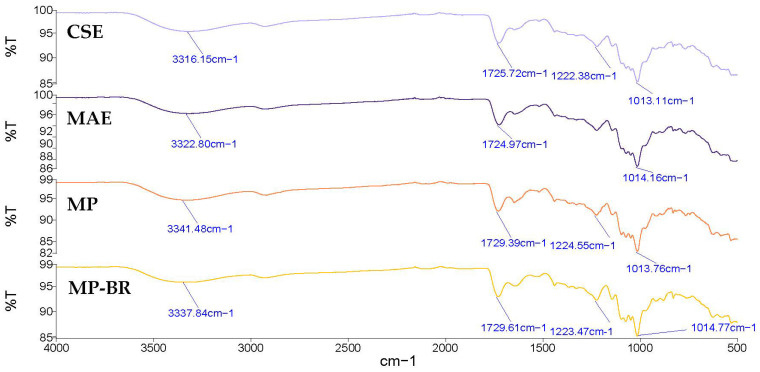
FTIR spectra of mandarin peel pectin extracted by different extraction methods. CSE—conventional solvent extraction; MAE—microwave-assisted extraction; MP—mandarin pectin obtained by optimized MAE from intact peel; MP-BR—mandarin pectin obtained using a biorefinery two-step process: optimized MAE of antioxidants from intact peel (extract 4, maximized all responses), followed by optimized MAE of pectin from the residue.

The FTIR spectra of the Satsuma mandarin peel pectin extracted by CSE and MAE are shown in Figure 8. Both spectra showed comparable characteristic bands, indicating a similar structural composition. The broad band appearing between 3600 and 3000 cm^−1^ refers to the O-H stretching vibration due to the inter- and intramolecular hydrogen bonding of the galacturonic acid polymer. The band around 2950 cm^−1^ refers to the C-H stretching vibration, including CH, CH_2_, and CH_3_ groups [71]. The band observed in the areas of 1730 cm^−1^ corresponded to the C=O stretching vibration of esterified carboxyl groups, and the band at 1600–1630 cm^−1^ was related to the C=O stretching vibration of free carboxyl groups [69]. Both pectin samples had band values lower than 1741 cm^−1^, which indicates that these pectin samples have low contents of hydrophobic groups [72]. The peaks at 1013 cm^−1^ and 1014 cm^−1^ refer to C–O, C–C and C–O–H stretching [73]. The area between 1200 cm^−1^ and 800 cm^−1^ is usually called the ‘fingerprint’ region, since the position and intensity of the individual band are unique for each polysaccharide and can be difficult to interpret [67]. Similar bands were observed in pectin extracted from Satsuma mandarin peel [25] and lime peel pectin [70].

In addition to the possibility of higher yields, green extraction methods, such as MAE, are usually claimed to have a lower ecological footprint compared to CSE. Even though this aspect is often emphasized, when pointing out the advantages of green extraction techniques, the comparison of the actual ecological footprint (or one aspect of it) of the green extraction with the CSE is rarely addressed in the literature. Such data are lacking for citrus pectin but recently Golub and co-workers [74] utilized LCA for comparing CSE and MAE of pectin extracted from tomato pomace and showed that one conventional extraction treatment has higher environmental impacts compared to one microwave extraction treatment for all the analyzed indicators (climate change, freshwater eutrophication, human toxicity, ionizing radiation and ozone depletion), more than twice as much. Similarly, Joglekar and co-workers [75] conducted LCA to compare MAE and conventional pectin extraction form citrus waste and proved the significantly lower ecological footprint of MAE.

In this work, we compared the energy consumption per g of the raw material of the two processes (MAE and CSE), and the obtained results are presented in Table 9. The presented results clearly show the significant advantages of MAE, showing four times lower energy consumption compared to CSE.

### 3.4. Two-Step MAE of Antioxidants and Pectin—Development of Biorefinery Approach

**Figure 9:**
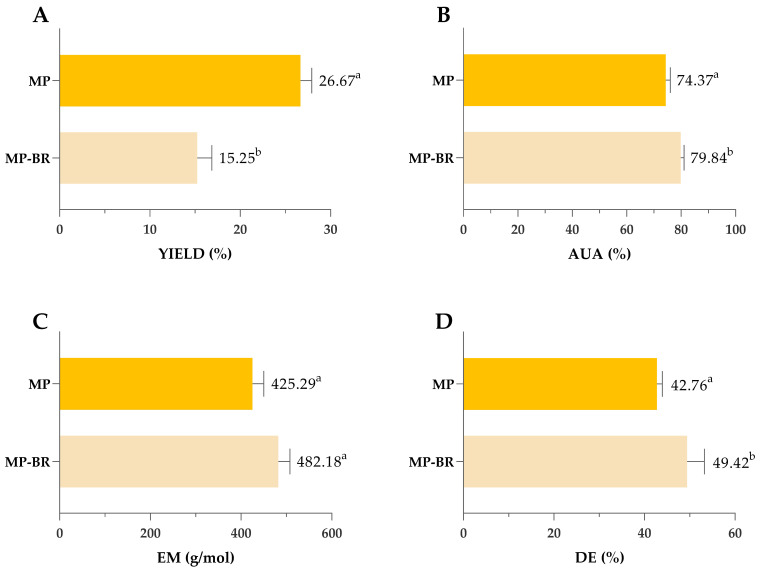
Comparison of yield (**A**), AUA (**B**), EM (**C**) and DE (**D**) of pectin obtained by MAE by integrated two-step procedure. MP—mandarin pectin obtained by optimized MAE from intact peel; MP-BR—mandarin pectin obtained using a biorefinery two-step process: optimized MAE of antioxidants from intact peel (extract 4, maximized all responses), followed by optimized MAE of pectin from the residue. Columns marked with different letters belong to different statistical groups (*p* < 0.05).

As mentioned in the Section 1, an additional goal of this research was to investigate the possibility of a biorefinery approach that would integrate the antioxidant and pectin extractions from mandarin peel into a single two-step process where pectin is extracted from the residue remaining after the extraction of antioxidants. Despite the general advantages of this approach, to our knowledge, such investigations have not been carried out with mandarin peel, so the effects on the quality and yield of pectin are unknown. The obtained results comparing yields and characteristics of mandarin pectin obtained by optimized MAE from intact peel (MP) and mandarin pectin obtained using a biorefinery two-step process—comprising optimized MAE of antioxidants from intact peel (extract 4, maximized all responses) followed by optimized MAE of pectin from the residue (MP-BR)—are shown in Figure 9.

**Figure 10:**
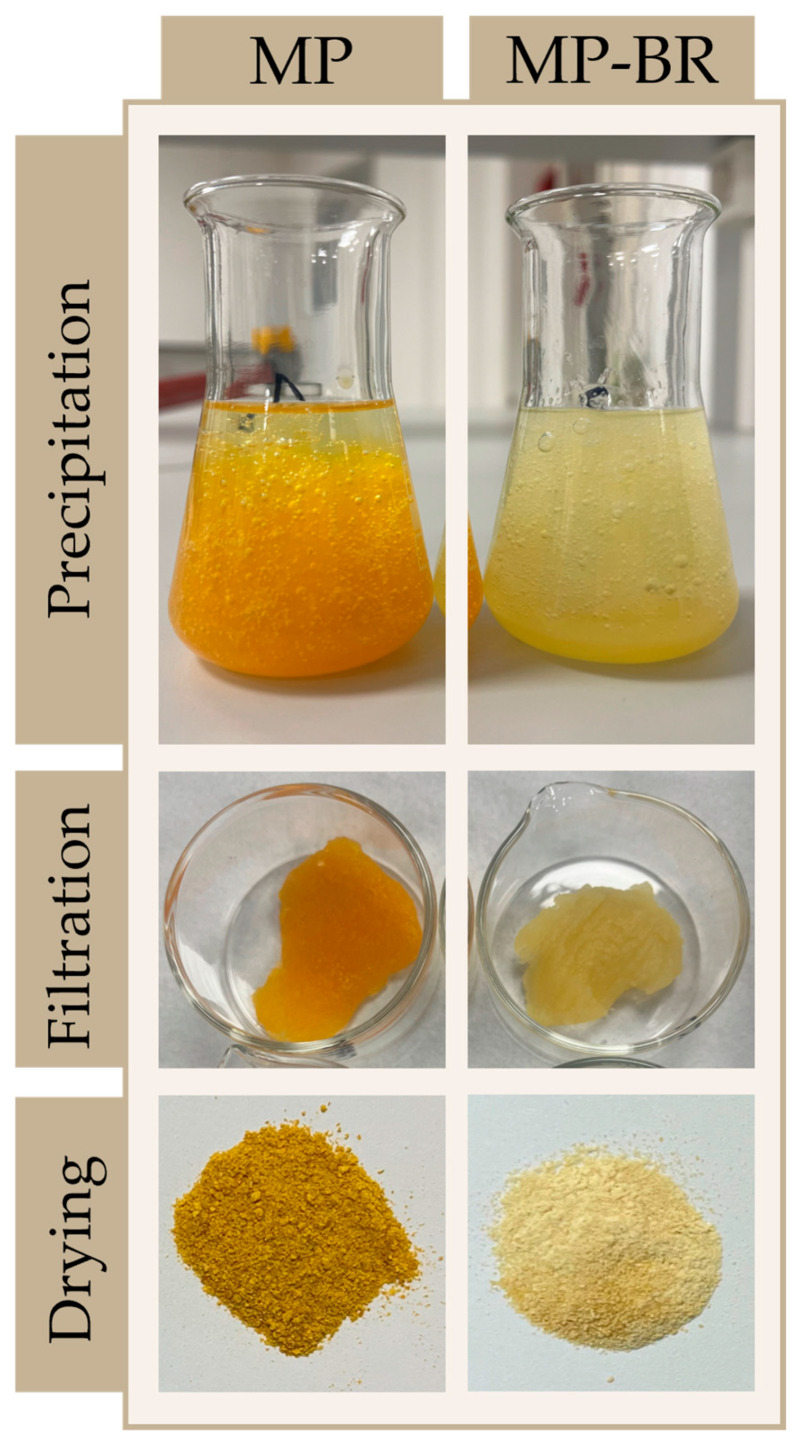
Comparison of the colour of mandarin peel pectin obtained by the single- or two-step extraction process. MP—mandarin pectin obtained by optimized MAE from intact peel; MP-BR—mandarin pectin obtained using a biorefinery two-step process: optimized MAE of antioxidants from intact peel (extract 4, maximized all responses), followed by optimized MAE of pectin from the residue.

As shown in Figure 9, sequential extraction of polyphenols and pectin resulted in a 12% reduction in pectin yield compared to non-sequential extraction. However, this reduction was accompanied by improvements in pectin quality, including higher AUA, EM and DE values and a lighter colour (Figure 10).

The obtained results are in line with our expectations. Namely, for the extraction of antioxidants, 77% EtOH was used. Ethanol is usually used for the purification of raw pectin to achieve market-quality pectin (higher purity, lighter colour) as it removes remaining non-pectin polysaccharides and pigments from the protein fraction. In this two-step process, such purification of the raw material is conducted prior to pectin extraction, resulting in a lower yield but higher purity. This is obvious from the comparison of the organoleptic characteristics (colour) of MP and MP-BR presented in Figure 10—the ethanol pretreatment of mandarin peel caused the removal of pigments/coloured compounds (regarded as impurities in pectin extraction), which led to the colour change.

Even though the yield of MP-BR was lower compared to MP, the obtained values are still in accordance with the typical yields reported by other authors that used a purification step in pectin extraction. Duan and co-authors [25] extracted pectin from Satsuma mandarin peel by conventional extraction and high hydrostatic pressure, with a purification step involving washing the pectin with absolute ethanol and acetone three times, and achieved yields in the range of 15.34–18.99%, which is comparable to the yield of MP-BR.

Figure 8 also presents the FTIR spectra of MP and MP-BR, showing similar transmission patterns to the CSE and MAE pectin already discussed in Section 3.3.2. This confirms that pectin was successfully extracted from Satsuma mandarin peel using all applied methods.

Despite the mentioned advantages, such approaches are still rarely investigated and, to our knowledge, have only been applied to other citrus species, such as orange peel for the extraction of polyphenols, pectin and essential oils [76,77], while data on mandarin are still lacking.

As mentioned previously, investigations of the biorefinery approach for obtaining valuable products from secondary raw materials are scarce and are non-existent for mandarin peel. However, Guandalini and co-workers [78] also developed a two-step ultrasound-assisted extraction process for the sequential extraction of phenolics and pectin from mango peel. As in our work, they used the residue remaining after ethanolic extraction of polyphenols to extract pectin and obtained satisfactory yields (8.6%) of high-purity pectin. Other authors applied different sequences of extraction procedures but still managed to obtain a satisfactory yield and quality of targeted compounds. Figueira and co-workers [79] developed a two-step continuous process for the extraction of pectin and hesperidin from orange peel using CSE, with pectin yields varying from 17.23% to 20.61% and 1% of high-purity hesperidin (84%) in the subsequent extraction process. Similarly, Talekar and co-workers [80] applied a similar approach and optimized a low-temperature hydrothermal process that yielded high-methoxyl, food-quality pectin (18.8–20.9%) and high yields of punicalagin-rich phenolics (10.6–11.8%), while the solid residue was subsequently used for bio-ethanol production. The literature data are in consistent with our results justifying the validity of the biorefinery approach.

The existing literature shows that introducing a single two-step extraction process additionally offers significant economic and environmental advantages compared to using two separate extraction processes. It maximizes the valorization of agro-industrial waste by recovering high-value compounds in a single workflow, which reduces raw material losses and increases overall product yield while minimizing water use and shortening the time of extraction [81].

## 4. Conclusions

According to the obtained results, mandarin peel as an agro-industrial waste has great potential as a source of carotenoids, polyphenols, flavonoids and pectin. The obtained results show that the major advantage of MAE is a significantly shorter extraction time and lower energy consumption (except in the case of the extraction of antioxidants at room temperature) compared to CSE, while the yields of target compounds are, in most cases, comparable. The application of integrated polyphenols and pectin extraction resulted in slightly reduced pectin yields but significant improvements of pectin quality compared to non-sequential extraction. Future investigations should focus on exploring scale-up possibilities and more comprehensive assessments of the environmental and economic benefits of integrated extraction processes.

## Figures and Tables

**Table 1:** Independent factors and their levels used in the design for the optimization of antioxidants.

Factor		Factor Level	
	−1	0	1
		Numeric Factors	
A: power (W)	400	600	800
B: solvent (%)	30	60	90
C: raw material (%)	0.1	0.2	0.3

**Table 2:** Independent factors and their levels used in the design for the optimization of pectin extraction.

Factor		Factor Level	
	−1	0	1
		Numeric Factors	
A: time (min)	1	3	5
B: power (W)	1000	1400	1800
C: pH	1	1.5	2

**Table 3:** Design of the experiment matrix for the microwave-assisted extraction of antioxidants and alongside responses.

	F1	F2	F3	R1	R2	R3	R4	R5
Run	Power (W)	Solvent (%)	Raw Material (%)	TCC (µg Beta-Carotene/g)	TPC (mg GAE/g)	TFC (mg QE/g)	ABTS (mmol TE/g)	DPPH (mmol TE/g)
1	600	60	0.2	71.98 ± 7.11	19.84 ± 0.89	74.06 ± 0.42	0.23 ± 0.02	0.03 ± 0.00
2	800	90	0.2	325.68 ± 4.79	12.31 ± 1.07	64.25 ± 1.58	0.31 ± 0.01	0.03 ± 0.00
3	600	60	0.2	75.94 ± 7.61	20.36 ± 1.60	73.46 ± 0.46	0.26 ± 0.02	0.03 ± 0.00
4	600	30	0.1	70.17 ± 7.07	21.10 ± 1.81	69.19 ± 2.45	0.27 ± 0.02	0.03 ± 0.00
5	800	30	0.2	48.68 ± 3.35	19.04 ± 0.43	70.59 ± 4.00	0.29 ± 0.02	0.03 ± 0.00
6	400	60	0.1	87.38 ± 2.99	21.83 ± 1.31	81.56 ± 3.33	0.37 ± 0.02	0.03 ± 0.00
7	600	30	0.3	45.57 ± 4.32	17.53 ± 0.87	62.61 ± 1.23	0.20 ± 0.01	0.02 ± 0.00
8	800	60	0.3	64.77 ± 2.48	20.00 ± 1.24	76.96 ± 3.38	0.29 ± 0.01	0.03 ± 0.00
9	600	60	0.2	60.47 ± 5.67	20.55 ± 1.10	79.62 ± 2.65	0.24 ± 0.02	0.03 ± 0.00
10	600	90	0.1	335.59 ± 10.79	19.29 ± 1.23	61.10 ± 0.81	0.25 ± 0.01	0.02 ± 0.00
11	800	60	0.1	84.14 ± 3.34	20.67 ± 0.74	71.95 ± 4.06	0.37 ± 0.02	0.03 ± 0.00
12	600	90	0.3	307.45 ± 14.17	15.94 ± 1.36	59.41 ± 0.98	0.19 ± 0.01	0.02 ± 0.00
13	400	90	0.2	352.39 ± 2.64	18.82 ± 0.98	73.83 ± 2.57	0.32 ± 0.01	0.02 ± 0.00
14	400	30	0.2	47.16 ± 2.50	19.13 ± 1.22	71.63 ± 1.70	0.31 ± 0.02	0.03 ± 0.00
15	400	60	0.3	70.04 ± 2.81	19.93 ± 1.17	82.94 ± 1.35	0.28 ± 0.01	0.02 ± 0.00
16	600	60	0.2	65.57 ± 2.99	20.94 ± 0.33	76.20 ± 0.17	0.25 ± 0.01	0.03 ± 0.00
17	600	60	0.2	77.73 ± 7.12	21.25 ± 1.43	77.77 ± 2.98	0.25 ± 0.02	0.03 ± 0.00

TCC—total carotenoid content; TPC—total phenolic content; TFC—total flavonoid content, ABTS—2,2-azino-bis (3-ethylbenzothiazoline-6-sulfonic acid) radical scavenging assay; DPPH—2,2-diphenyl-1-picrylhydrazyl radical scavenging assay; GAE—gallic acid equivalents; QE—quercetin equivalents; TE—Trolox equivalents.

**Table 4:** Optimal yields of polyphenols, carotenoids and antioxidants depending on the target compound (extract 1–extract 4) and comparison of predicted and experimental data.

	TCC (µg β-Carotene/g d.w.)	TFC (mg QE/g d.w.)	TPC (mg GAE/g d.w.)	DPPH (mmol TE/g d.w.)	ABTS (mmol TE/g d.w.)	Desirability
Predicted TCC	354.064					1.000
Predicted TFC/TPC		83.283	21.878			1.000
Predicted DPPH/ABTS				0.032	0.367	0.991
Predicted TCC/TFC/TPC/DPPH/ABTS	201.245	80.191	20.803	0.025	0.335	0.711
Experimental TCC	352.267 ± 17.402					
Experimental FC/TPC		83.128 ± 1.979	21.764 ± 0.456			
Experimental DPPH/ABTS				0.033 ± 0.001	0.366 ± 0.016	
Experimental TCC/TFC/TPC/DPPH/ABTS	189.230 ± 16.498	77.050 ± 1.557	21.230 ± 0.993	0.026 ± 0.001	0.244 ± 0.010	
Predicted vs. experimental 1 (%)	0.508					
Predicted vs. experimental 2 (%)		0.186	0.521			
Predicted vs. experimental 3 (%)				−3.125	0.272	
Predicted vs. experimental 4 (%)	5.971	3.917	−2.053	−4.000	27.164	

TCC (raw material-0.11%/power-400 W/ethanol-90%); TFC/TPC (raw material-0.14%/power-400 W/ethanol-54%); DPPH/ABTS (raw material-0.10%/power-400 W/ethanol-41%); TCC/TFC/TPC/DPPH/ABTS (raw material-0.16%/power-400 W/ethanol 77%). Time of MAE in all optimal extracts-4 min.

**Table 5:** Content of nobiletin and tangeretin in optimal extracts.

Optimal Conditions	Nobiletin (µg/g d.w.)	Tangeretin (µg/g d.w.)
Extract 1	703.62 ± 51.72 ^a^	134.22 ± 8.96 ^a^
Extract 2	570.47 ± 25.00 ^b^	138.24 ± 4.65 ^a^
Extract 3	592.00 ± 4.86 ^b^	139.7 ± 2.28 ^a^
Extract 4	610.07 ± 2.06 ^ab^	135.24 ± 0.51 ^a^

Extract 1—maximized total carotenoid content(0.11% raw material/400 W power/90% ethanol); extract 2—maximized total phenolic and flavonoid content (0.14% raw material/400 W power/54% ethanol); extract 3—maximized antioxidative activity (0.14% raw material/400 W power/54% ethanol); extract 4—maximized all responses (0.16% raw material/400 W power/77% ethanol). Results in the same column marked with different letters belong to different statistical groups (*p* < 0.001).

**Table 6:** Design of the experiment matrix for the microwave-assisted extraction of pectin and alongside responses.

Run	F1	F2	F3	R1	R2	R3
	Time (min)	Power (W)	pH	Yield (%)	AUA (%)	DE (%)
1	3	1400	1.5	24.26 ± 3.46	59.74 ± 0.24	47.84 ± 0.62
2	3	1000	2	9.23 ± 0.54	55.70 ± 0.05	62.76 ± 1.25
3	5	1800	1.5	7.89 ± 1.04	60.52 ± 0.71	64.56 ± 0.41
4	5	1400	2	23.47 ± 0.94	59.39 ± 1.17	54.03 ± 0.75
5	3	1400	1.5	24.97 ± 1.33	63.11 ± 0.72	43.91 ± 0.75
6	3	1800	1	19.57 ± 1.19	78.84 ± 0.74	39.28 ± 0.35
7	3	1000	1	18.07 ± 1.09	61.25 ± 2.28	53.78 ± 1.78
8	1	1800	1.5	10.53 ± 0.18	60.97 ± 3.38	61.50 ± 1.92
9	3	1400	1.5	23.07 ± 2.27	66.83 ± 1.55	49.53 ± 1.19
10	1	1000	1.5	6.42 ± 0.02	64.88 ± 3.39	61.96 ± 1.14
11	3	1800	2	22.57 ± 2.43	63.86 ± 2.53	53.64 ± 2.46
12	1	1400	1	11.32 ± 0.86	64.25 ± 1.59	54.20 ± 0.86
13	3	1400	1.5	24.49 ± 3.85	68.96 ± 3.21	46.13 ± 3.30
14	3	1400	1.5	25.16 ± 1.64	65.76 ± 1.51	47.59 ± 0.93
15	5	1000	1.5	24.93 ± 0.67	65.99 ± 0.85	48.87 ± 0.45
16	1	1400	2	4.19 ± 0.17	60.15 ± 1.17	68.39 ± 2.45
17	5	1400	1	11.57 ± 1.23	78.69 ± 1.02	38.20 ± 0.38

AUA—anhydrouronic acid content; DE—degree of esterification.

**Table 7:** Predicted vs. experimental values of target parameters under optimal MAE conditions for pectin extraction.

	Yield (%)	AUA (%)	DE (%)
Predicted	25.072	65	51.089
Experimental	27.277	71.525	42.354
Predicted vs. experimental (%)	−8.794	−10.038	−20.623

AUA—anhydrouronic acid content; DE—degree of esterification.

**Table 8:** Comparison of CSE and MAE regarding the yields of antioxidants under the conditions optimized for extraction 4.

Extraction Method	Content of Observed Outputs (Extract 4)						
	TPC (mg GAE/g d.w.)	TFC (mg QE/g d.w.)	TCC (µg β-Carotene/g d.w.)	ABTS (µmol TE/g d.w.)	DPPH (µmol TE/g d.w.)	Nobiletin (µg/g d.w.)	Tangeretin (µg/g d.w.)
CSE	28.24 ± 1.16 ^a^	82.91 ± 1.07 ^a^	214.11 ± 15.49 ^a^	189.61 ± 2.81 ^a^	32.15 ± 1.15 ^a^	881.28 ± 12.13 ^a^	158.23 ± 2.30 ^a^
MAE	21.23 ± 0.99 ^b^	77.05 ± 1.56 ^b^	189.21 ± 16.50 ^a^	244.21 ± 9.69 ^b^	26.07 ± 0.53 ^b^	610.07 ± 2.06 ^b^	135.24 ± 0.51 ^b^

Extract 4—maximized all responses (0.16% raw material/400 W power/77% ethanol); TPC—total phenolic content; TFC—total flavonoid content; TCC—total carotenoid content; CSE—conventional solvent extraction; MAE—microwave-assisted extraction; ABTS—2,2-azino-bis (3-ethylbenzothiazoline-6-sulfonic acid) radical scavenging assay; DPPH—2,2-diphenyl-1-picrylhydrazyl radical scavenging assay; GAE—gallic acid equivalents; QE—quercetin equivalents; TE—Trolox equivalents. Results in the same column marked with different letters belong to different statistical groups (*p* < 0.001).

**Table 9:** Comparison of the extraction times and energy consumption for the MAE and CSE preparation of extracts under optimized conditions.

	Energy Consumption (kWh/kg)		Time (min)	
	CSE	MAE	CSE	MAE
Extract 1	162	32.3	30	4
Extract 2	286	25.4	30	4
Extract 3	0.05	35.6	30	4
Extract 4	374	22.2	30	4
Pectin extract	29.6	2.2	120	3.7

CSE—conventional solvent extraction; MAE—microwave-assisted extraction; extract 1—raw material-0.11%/power-400 W/ethanol-90%; extract 2—raw material-0.14%/power-400 W/ethanol-54%; extract 3—raw material-0.10%/power-400 W/ethanol-41%; extract 4—raw material-0.16%/power-400 W/ethanol-77%.

## Data Availability

The original contributions presented in this study are included in the article/Supplementary Materials. Further inquiries can be directed to the corresponding author.

## Supplementary Materials

The following supporting information can be downloaded at: https://www.mdpi.com/article/10.3390/antiox14060722/s1, Figure S1: HPLC chromatograms at 280 nm and 330 nm.; Figure S2: Response surface plots (3D) for pectin as a function of power, time and pH; Figure S3. Normal plots of residuals for A (TCC), B (TPC), C (TFC), D (ABTS) and E (DPPH); Table S1: The model equations for the CSE of antioxidants; Table S2: The model equations for the MAE of antioxidants and pectin; Table S3. ANOVA for response surface quadratic model for antioxidants; Table S4. ANOVA for response surface quadratic model for pectin.

## Abbreviations

The following abbreviations are used in this manuscript:

AUA	Anhydrouronic acid content
ABTS	2,2-azino-bis (3-ethylbenzothiazoline-6-sulfonic acid) radical scavenging assay
CSE	Conventional solvent extraction
DAD	Diode-array detector
DE	Degree of esterification
DPPH	2,2-diphenyl-1-picrylhydrazyl radical scavenging assay
EM	Equivalent mass
FTIR	Fourier transform infrared spectroscopy
GAE	Gallic acid equivalents
HPLC	High-performance liquid chromatography
MAE	Microwave-assisted extraction
MC	Methoxyl content
MP	Mandarin pectin obtained by optimized MAE from intact peel
MP-BR	Mandarin pectin obtained using a biorefinery two-step process: optimized MAE of antioxidants from intact peel (extract 4, maximized all responses), followed by optimized MAE of pectin from the residue
PMFs	Polymethoxyflavones
QE	Quercetin equivalents
RSM	Response surface methodology
TCC	Total carotenoid content
TE	Trolox equivalents
TFC	Total flavonoid content
TPC	Total phenolic content
UAE	Ultrasound-assisted extraction
