# Supplementary material for: Microwave-Assisted Extraction of Bioactive Compounds from Mandarin Peel: A Comprehensive Biorefinery Strategy
Source: Antioxidants (Basel). 2025 Jun 12;14(6):722. doi: 10.3390/antiox14060722 (PMC12189523; doi:10.3390/antiox14060722)
Supplement: Supplementary file 1 [file antioxidants-14-00722-s001.zip › Table S1.pdf]

Table S1. The model equations for the CSE of antioxidants

	Final equations in terms of coded factors - antioxidants
TCC	TCC =+63.63+10.04xA+31.31xB+177.52xC+17.47xAxB-3.24xAxC+23.48x BxC+9.58xA2-2.62xB2+155.02xC2
TPC	TPC = +27.20 -1.92xA +4.97xB +7.33xC -0.18xAxB +0.33xAxC +2.28xBxC -0,49x A2 -0.65xB2 +3.89xC2
TFC	TFC = +77.68 -1.56xA +9.52xB -3.48xC +0.10xAxB+2.01xAxC -0.71xBxC -3.39x A2 +2.15xB2 -15.17xC2
ABTS	ABTS = +0.18 -0.031xA +9.743E-003xB -8.222E-003xC -1.353E-003xAxB+7.953E-003xAxC +4.839E-003xBxC -3.209E-004xA2 +0.021xB2 -0.020xC2
DPPH	DPPH =+0.026+1.240E-003×A-2.569E-003×B-2.157E-003×C+8.075E-004×A× B+2.088E-003×A×C+2.507E-003×B× C+1.313E-003×A2+3.566E-004×B2-1.860E-003×C2

TCC—total carotenoid content; TPC—total phenolic content; TFC—total flavonoid content.
