# Supplementary material for: Microwave-Assisted Extraction of Bioactive Compounds from Mandarin Peel: A Comprehensive Biorefinery Strategy
Source: Antioxidants (Basel). 2025 Jun 12;14(6):722. doi: 10.3390/antiox14060722 (PMC12189523; doi:10.3390/antiox14060722)
Supplement: Supplementary file 1 [file antioxidants-14-00722-s001.zip › Table S2.pdf]

Table S2. The model equations for the MAE of antioxidants and pectin.

	Final equations in terms of coded factors - antioxidants
TCC	TCC =+70.34 -11.18×A +138.69× B-4.21×C-0.88×A× B-0.51×A× C-7.06× B× C+1.23×A^2+118.13× B^2+5.01×C^2
TPC	TPC =+20.59-1.19×A-1.31×B-0.96×C+0.055×A×B+0.31×A× C-1.61×B×C+0.58×A^2-2.70×B^2-0.56×C^2
TFC	TFC =+76.22-0.23×A-1.93×B-3.27×C+1.22×A× B+0.91×A× C-2.14 × B×C-2.43×A^2-10.71×B^2+4.56×C^2
ABTS	ABTS =+0.25-0.039 ×A-8.123E-004×B-2.347E-003 ×C+ 2.659E-003 ×A× B+2.157E-003×A× C+ 2.502E-003 × B×C-1.332E-004 ×A^2-0.018×B^2 +0.081 ×C^2
DPPH	DPPH =+0.026- 2.157E-003 ×A-2.569E-003×B+1.240E-003 ×C+ 2.507E-003 ×A× B+ 2.088 E-003 ×A×C+8.075E-004×B× C-1.860E-003×A^2+3.566E-004×B^2+1.313E-003×C^2
	**Final equations in terms of coded factors - pectin**
YIELD	y =24.39+4.43×A+0.2409×B-0.1336× C-5.29×AB+4.76×AC +2.96×BC-8.34×A^2-3.61×B^2-3.42×C^2
AUA	y= 64.64+1.79×A+2.05×B -5.49×C
DE	y=52.72-5.05×A-1.05×B+6.67×C

TCC—total carotenoid content; TPC—total phenolic content; TFC—total flavonoid content; AUA—anhydrouronic acid content; DE—degree of esterification.
