# Supplementary material for: Microwave-Assisted Extraction of Bioactive Compounds from Mandarin Peel: A Comprehensive Biorefinery Strategy
Source: Antioxidants (Basel). 2025 Jun 12;14(6):722. doi: 10.3390/antiox14060722 (PMC12189523; doi:10.3390/antiox14060722)
Supplement: Supplementary file 1 [file antioxidants-14-00722-s001.zip › Table S3.pdf]

Table S3. ANOVA for Response Surface Quadratic Model for antioxidants

Response	Source	Sum of Squares	df	Mean Square	F-value	p-value Prob > F	Other
TCC	Model	2.148E+005	9	23865.63	255.90	< 0.0001	Std. Dev. = 9.66
	A-Raw material	1000.07	1	1000.07	10.72	0.0136	Mean = 128.87
	B-Solvent	1.539E+005	1	1.539E+005	1650.04	< 0.0001	C.V. % = 7.49
	C-Power	142.02	1	142.02	1.52	0.2570	
	AB	3.11	1	3.11	0.033	0.8602	R2 = 0.9970
	AC	1.03	1	1.03	0.011	0.9193	Adj R2 = 0.9931
	BC	199.25	1	199.25	2.14	0.1872	
	A2	6.40	1	6.40	0.069	0.8009	Adeq Precision = 40.873
	B2	58752.16	1	58752.16	629.97	< 0.0001	
	C2	105.77	1	105.77	1.13	0.3223	
	Residual	652.83	7	93.26			
	Lack of Fit	444.14	3	148.05	2.84	0.1698	
	Pure Error	208.69	4	52.17			
	Cor Total	2.154E+005	16				
TPC	Model	76.50	9	8.50	6.63	0.0104	Std. Dev. = 1.13
	A-Raw material	11.27	1	11.27	8.78	0.0210	Mean = 19.33
	B-Solvent	13.63	1	13.63	10.62	0.0139	C.V. % = 5.86
	C-Power	7.38	1	7.38	5.75	0.0475	
	AB	0.012	1	0.012	9.276E-003	0.9260	R2 = 0.8949
	AC	0.38	1	0.38	0.30	0.6023	Adj R2 = 0.7599
	BC	10.31	1	10.31	8.03	0.0252	
	A2	1.42	1	1.42	1.11	0.3275	Adeq Precision = 11.064
	B2	30.76	1	30.76	23.97	0.0018	
	C2	1.32	1	1.32	1.03	0.3439	
	Residual	8.98	7	1.28			
	Lack of Fit	7.81	3	2.60	8.91	0.0304	
	Pure Error	1.17	4	0.29			
	Cor Total	85.49	16				
TFC	Model	726.53	9	80.73	9.03	0.0042	Std. Dev. = 2.99
	A-Raw material	0.44	1	0.44	0.049	0.8306	Mean = 72.18
	B-Solvent	29.71	1	29.71	3.32	0.1111	C.V. % = 4.14
	C-Power	85.74	1	85.74	9.59	0.0174	
	AB	5.98	1	5.98	0.67	0.4404	R2 = 0.9207
	AC	3.31	1	3.31	0.37	0.5620	Adj R2 = 0.8187
	BC	18.25	1	18.25	2.04	0.1962	
	A2	24.94	1	24.94	2.79	0.1388	Adeq Precision = 9.858
	B2	482.94	1	482.94	54.02	0.0002	
	C2	87.67	1	87.67	9.81	0.0166	
	Residual	62.57	7	8.94			
	Lack of Fit	36.35	3	12.12	1.85	0.2790	
	Pure Error	26.23	4	6.56			
	Cor Total	789.10	16				

Response	Source	Sum of Squares	df	Mean Square	F-value	p-value Prob > F	Other
ABTS	Model	0.041	9	4.544E-003	22.89	0.0002	Std. Dev. = 0.014
	A-Raw material	0.012	1	0.012	60.83	0.0001	Mean = 0.28
	B-Solvent	5.279E-006	1	5.279E-006	0.027	0.8751	C.V. % = 5.12
	C-Power	4.406E-005	1	4.406E-005	0.22	0.6519	
	AB	2.828E-005	1	2.828E-005	0.14	0.7170	R^2 = 0.9671
	AC	1.862E-005	1	1.862E-005	0.094	0.7683	Adj R^2 = 0.9249
	BC	2.504E-005	1	2.504E-005	0.13	0.7329	
	A2	7.472E-008	1	7.472E-008	3.765E-004	0.9851	Adeq Precision = 16.953
	B2	1.327E-003	1	1.327E-003	6.69	0.0362	
	C2	0.028	1	0.028	140.48	< 0.0001	
	Residual	1.389E-003	7	1.985E-004			
	Lack of Fit	9.626E-004	3	3.209E-004	3.01	0.1576	
	Pure Error	4.268E-004	4	1.067E-004			
	Cor Total	0.042	16				
DPPH	Model	1.688E-004	9	1.875E-005	8.72	0.0047	Std. Dev. = 1.467E-003
	A-Raw material	3.721E-005	1	3.721E-005	17.30	0.0042	Mean = 0.026
	B-Solvent	5.282E-005	1	5.282E-005	24.56	0.0016	C.V. % = 5.61
	C-Power	1.231E-005	1	1.231E-005	5.72	0.0480	
	AB	2.514E-005	1	2.514E-005	11.69	0.0112	R^2 = 0.9181
	AC	1.743E-005	1	1.743E-005	8.10	0.0248	Adj R^2 = 0.8128
	BC	2.608E-006	1	2.608E-006	1.21	0.3072	
	A2	1.457E-005	1	1.457E-005	6.77	0.0353	Adeq Precision = 10.457
	B2	5.353E-007	1	5.353E-007	0.25	0.6332	
	C2	7.254E-006	1	7.254E-006	3.37	0.1089	
	Residual	1.506E-005	7	2.151E-006			
	Lack of Fit	1.434E-005	3	4.781E-006	26.84	0.0041	
	Pure Error	7.125E-007	4	1.781E-007			
	Cor Total	1.838E-004	16				
