# Supplementary material for: Microwave-Assisted Extraction of Bioactive Compounds from Mandarin Peel: A Comprehensive Biorefinery Strategy
Source: Antioxidants (Basel). 2025 Jun 12;14(6):722. doi: 10.3390/antiox14060722 (PMC12189523; doi:10.3390/antiox14060722)
Supplement: Supplementary file 1 [file antioxidants-14-00722-s001.zip › Table S4.pdf]

**Table S4.** ANOVA for Response Surface Quadratic Model for pectin

| Response | Source | Sum of Squares | df | Mean Square | F-value | p-value | Other |
|---|---|---|---|---|---|---|---|
| Yield | Model | 827.11 | 9 | 91.9 | 5.6 | 0.0167 | Std. Dev. = 4.05 |
| | A-Time | 156.72 | 1 | 156.72 | 9.54 | 0.0176 | Mean = 17.16 |
| | B-Power | 0.4644 | 1 | 0.4644 | 0.0283 | 0.8712 | C.V. % = 23.62 |
| | C-pH | 0.1428 | 1 | 0.1428 | 0.0087 | 0.9283 | $R^2$ = 0.878 |
| | AB | 111.9 | 1 | 111.9 | 6.82 | 0.0349 | Adjusted $R^2$ = 0.7211 |
| | AC | 90.47 | 1 | 90.47 | 5.51 | 0.0513 | Adeq Precision = 7.0481 |
| | BC | 35.04 | 1 | 35.04 | 2.13 | 0.1875 | |
| | $A^2$ | 292.6 | 1 | 292.6 | 17.82 | 0.0039 | |
| | $B^2$ | 54.92 | 1 | 54.92 | 3.34 | 0.1101 | |
| | $C^2$ | 49.14 | 1 | 49.14 | 2.99 | 0.1272 | |
| | Residual | 114.93 | 7 | 16.42 | | | |
| | Lack of Fit | 112.24 | 3 | 37.41 | 55.6 | 0.001 | |
| | Pure Error | 2.69 | 4 | 0.6729 | | | |
| | Cor Total | 942.04 | 16 | | | | |
| AUA | Model | 300.49 | 3 | 100.16 | 4.05 | 0.031 | Std. Dev. = 4.98 |
| | A-Time | 25.7 | 1 | 25.7 | 1.04 | 0.3269 | Mean 64.64 |
| | B-Power | 33.52 | 1 | 33.52 | 1.35 | 0.2655 | C.V. % = 7.7 |
| | C-pH | 241.26 | 1 | 241.26 | 9.74 | 0.0081 | $R^2$ = 0.4828 |
| | Residual | 321.89 | 13 | 24.76 | | | Adjusted $R^2$ = 0.3635 |
| | Lack of Fit | 271.15 | 9 | 30.13 | 2.37 | 0.2101 | Adeq Precision = 6.2465 |
| | Pure Error | 50.74 | 4 | 12.69 | | | |
| | Cor Total | 622.38 | 16 | | | | |
| DE | Model | 568.68 | 3 | 189.56 | 3.66 | 0.0414 | Std. Dev. = 7.2 |
| | A-Time | 203.89 | 1 | 203.89 | 3.93 | 0.0688 | Mean = 52.72 |
| | B-Power | 8.8 | 1 | 8.8 | 0.1699 | 0.6869 | C.V. % = 13.66 |
| | C-pH | 355.99 | 1 | 355.99 | 6.87 | 0.0211 | $R^2$ = 0.4577 |
| | Residual | 673.71 | 13 | 51.82 | | | Adjusted $R^2$ = 0.3326 |
| | Lack of Fit | 655.97 | 9 | 72.89 | 16.43 | 0.0081 | Adeq Precision = 6.7121 |
| | Pure Error | 17.74 | 4 | 4.44 | | | |
| | Cor Total | 1242.39 | 16 | | | | |
