# Supplementary figures and images for: Microwave-Assisted Extraction of Bioactive Compounds from Mandarin Peel: A Comprehensive Biorefinery Strategy
Source: Antioxidants (Basel). 2025 Jun 12;14(6):722. doi: 10.3390/antiox14060722 (PMC12189523; doi:10.3390/antiox14060722)

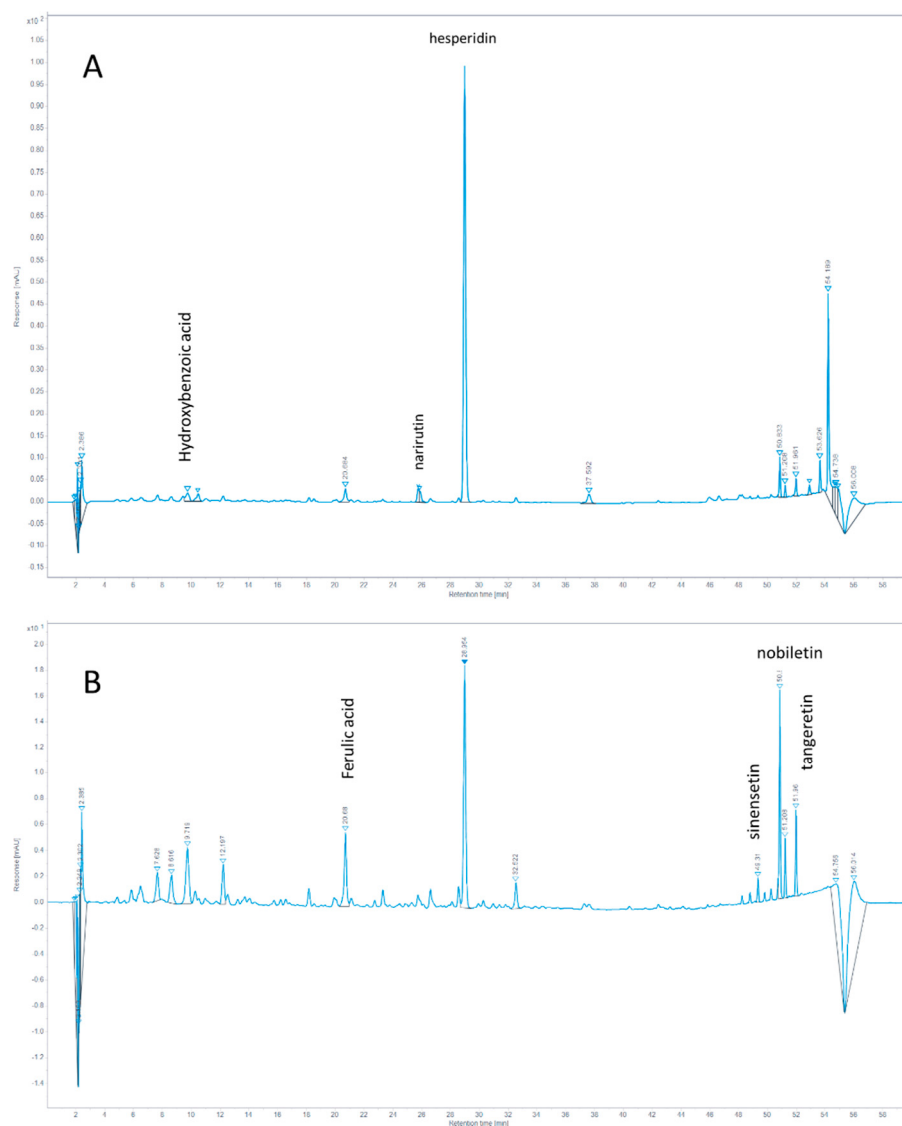

**Figure S1.** HPLC chromatograms at 280 nm (A) and 330 nm (B).

Supplement: Supplementary file 1 [file antioxidants-14-00722-s001.zip › Figure S1.pdf]

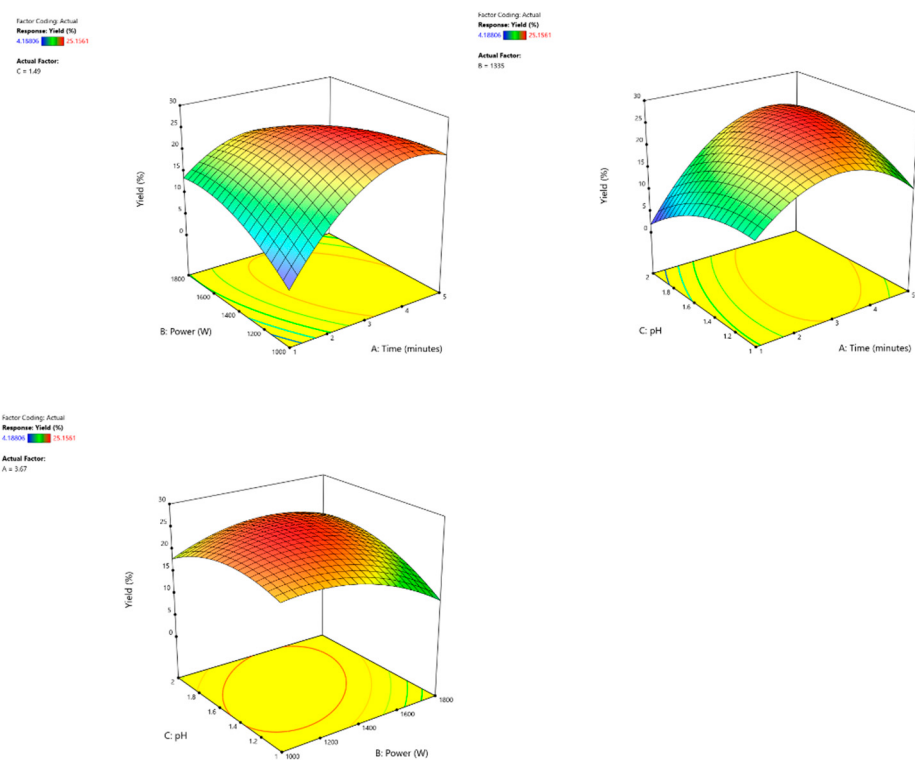

**Figure S2** Response surface plots (3D) for pectin as a function of power, time, and pH.

Supplement: Supplementary file 1 [file antioxidants-14-00722-s001.zip › Figure S2.pdf]

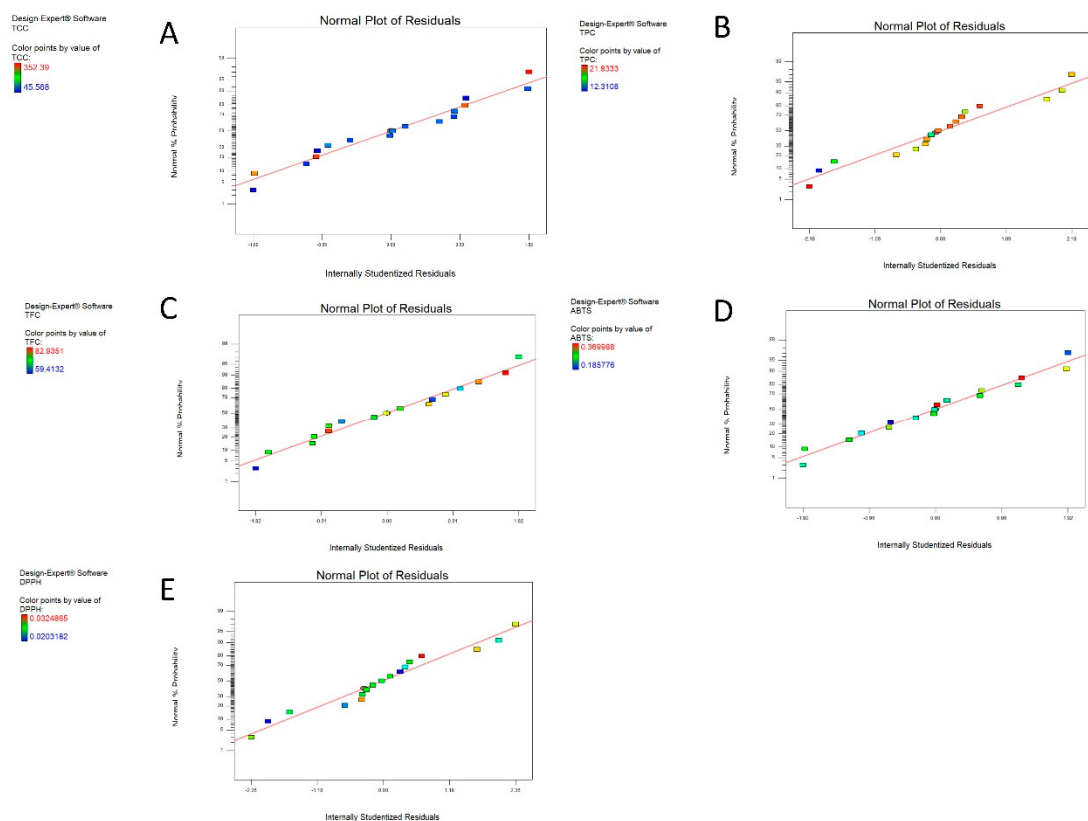

Figure S3. Normal plots of residuals for A (TCC), B (TPC), C (TFC), D (ABTS), E (DPPH).

Supplement: Supplementary file 1 [file antioxidants-14-00722-s001.zip › Figure S3.pdf]
